# Carriers of the p.P522R variant in PLCγ2 have a slightly more responsive immune system

**DOI:** 10.1186/s13024-023-00604-9

**Published:** 2023-04-20

**Authors:** Annieck M. Diks, Cristina Teodosio, Bas de Mooij, Rick J. Groenland, Brigitta A. E. Naber, Inge F. de Laat, Sandra A. Vloemans, Susan Rohde, Marien I. de Jonge, Linda Lorenz, Debbie Horsten, Jacques J. M. van Dongen, Magdalena A. Berkowska, Henne Holstege

**Affiliations:** 1 Department of Immunology, Leiden University Medical Center, Albinusdreef 2, Leiden, ZA 2333 the Netherlands; 2 Department of Human Genetics, Vrije Universiteit Amsterdam, Amsterdam UMC, Amsterdam, the Netherlands; 3 Translational and Clinical Research Program, Cancer Research Center (IBMCC; University of Salamanca - CSIC), Salamanca, Spain; 4 Department of Medicine, University of Salamanca and Institute of Biomedical Research of Salamanca (IBSAL), Salamanca, Spain; 5 Laboratory of Medical Immunology, Radboud Institute for Molecular Life Sciences, Radboud University Medical Centre, Nijmegen, The Netherlands

**Keywords:** Phospholipase C gamma 2, PLCγ2 p.P522R, PLCG2 rs72824905, Healthy aging, Immunosenescence, Flow cytometry, Functional studies

## Abstract

**Background:**

The rs72824905 single-nucleotide polymorphism in the *PLCG2* gene, encoding the p.P522R residue change in Phospholipase C gamma 2 (PLCγ2), associates with protection against several dementia subtypes and with increased likelihood of longevity. Cell lines and animal models indicated that p.P522R is a functional hypermorph. We aimed to confirm this in human circulating peripheral immune cells.

**Methods:**

We compared effects of p.P522R on immune system function between carriers and non-carriers (aged 59-103y), using in-depth immunophenotyping, functional B-cell and myeloid cell assays, and in vivo SARS-CoV-2 vaccination.

**Results:**

In line with expectations, p.P522R impacts immune cell function only slightly, but it does so across a wide array of immune cell types. Upon B-cell stimulation, we observed increased PLCγ2 phosphorylation and calcium release, suggesting increased B-cell sensitivity upon antigen recognition. Further, p.P522R-carriers had higher numbers of CD20++CD21-CD24+ naive B cells and IgG1+ memory B cells. In myeloid cells, normalized ROS production was higher upon PLCγ2-dependent stimulation. On classical monocytes, CD33 levels were elevated. Furthermore, carriers expressed lower levels of allergy-related FcεRI on several immune cell subsets. Nevertheless, carriers and non-carriers had similar serological responses to SARS-CoV-2 vaccination.

**Conclusion:**

The immune system from p.P522R-carriers is slightly more responsive to stimulation than in non-carriers.

**Supplementary Information:**

The online version contains supplementary material available at 10.1186/s13024-023-00604-9.

## Background

The average human lifespan has greatly expanded in the past decennia. In most individuals, increase in age coincides with a decline in cognitive and physical health. Many age-related impairments, such as lowered resistance to infection, dementia, osteoporosis, atherosclerosis, and diabetes are directly or indirectly related to the aging immune system, especially to the low grade inflammation that is frequently observed in older individuals (inflamm-aging) [1, 2].

Upon aging, the output of naive B and T cells from the bone marrow into the periphery decreases, leading to lower numbers of naive lymphocytes in the circulation. Additionally, B and T-cell receptor (BCR, TCR) diversity decreases upon aging, leaving aged individuals less equipped to deal with neo-antigens [1, 3, 4]. Human and animal models have shown impaired germinal center (GC) formation, reduced affinity maturation, reduced memory B-cell (MBC) differentiation and lowered levels of plasma cells in the bone marrow [3]. Additionally, the functioning of innate immune cells declines. In neutrophils derived from aged individuals, the capacity to phagocytose opsonized particles was lowered, and production of reactive oxygen species (ROS) was decreased [4, 5]. Likewise, monocytes derived from older adults showed reduced phagocytic capacity, lowered ROS production and lowered anti-tumor properties [1, 6, 7]. However, while a decline of immune system function heavily affects the health of some individuals, others maintain a functioning immune system until extreme ages [8].

Previous studies found that carrying a single-nucleotide polymorphism (SNP) in the immune gene Phospholipase C gamma 2 (*PLCG2*) (p.P522R, rs72824905) is associated with optimally maintained physical and cognitive functions during the aging process [9–11]. In fact, this genetic variant was enriched in a cohort of cognitively healthy centenarians [10].

PLCγ2 is an enzyme with a critical regulatory role in various immune and inflammatory pathways. PLCγ2 is involved in downstream receptor signaling where, upon receptor stimulation, it hydrolyses PIP2 (phosphatidylinositol 4,5-bisphosphate) to generate IP3 (inositol 1,4,5-triphosphate) and DAG (diacylglycerol), which are second messenger molecules that further transmit the activation signal, leading to the release of intracellular calcium [12, 13]. Amongst others, PCLγ2 is expressed downstream of several members of the immunoglobulin superfamily receptors, such as the BCR on B cells, but also downstream of Fc receptors (FcR) in innate cells, such as FcRγIII and 2B4 on NK cells, FcεRI on mast cells, TREM (Triggering Receptors Expressed on Myeloid cells) on macrophages and microglia, FcR-involving collagen receptors on platelets, and downstream of Toll-like receptors [13–16].

The p.P522R variant was not only associated with increased likelihood of extreme longevity, but also with a lower risk of several forms of age-related neurodegenerative diseases, including Alzheimer’s disease, frontotemporal dementia, dementia with Lewy Bodies, and progressive supranuclear palsy [10, 17]. Recently, multiple sclerosis was added to this list [18]. A recent study suggested that the p.P522R variant may maintain cognitive function during aging by mitigating the accumulation of toxic tau pathology in the brain, when this co-occurs with the accumulation of amyloid pathology, both hallmarks of Alzheimer’s disease [19]. The same study suggested that the cognitive decline observed in individuals who carry the *APOE-ε4* allele, the strongest genetic risk factor for Alzheimer’s disease, in combination with the p.P522R *PLCG2* variant, was slower and less pronounced compared to individuals who carried the *APOE-ε4* allele without the protective p.P522R variant [10, 19]. In fact, carrying the p.P522R *PLCG2* variant may have contributed to maintaining brain function in a 104-year-old cognitively healthy female, who is homozygous for the *APOE-ε4* allele [10]. Together, this suggests that the effect of p.P522R associates with maintaining an aspect of the immune system that, when compromised, increases the risk of several neurodegenerative diseases.

Studies in transfected COS7, HEK239T and BV2 cells, a p.P522R mouse model, and gene-edited human iPSCs suggested that p.P522R is a gain-of-function variant (functional hypermorph) [14, 20, 21]. Moreover, many studies have investigated the effect of the p.P522R *PLCG2* variant in the innate immune cells of the brain, the microglia. However, *PLCG2* is most prominently expressed in peripheral immune cells, suggesting that genetic variants in this gene may also affect human peripheral immune system function. Yet, an in-depth evaluation of the effect of p.P522R on the function of circulating immune cells has not yet been performed. Here, we investigated the quantities, immunophenotypes, and functions of circulating immune cells in p.P522R-carriers and non-carriers from 9 different nuclear families, in which one parent reached 100 years with high levels of cognitive performance and carried the p.P522R variant. We evaluated phosphorylation of PLCγ2 and calcium release upon BCR stimulation and analyzed the replication history in B-cell subsets. Furthermore, we monitored phagocytosis and ROS production in myeloid cell subsets upon exposure to opsonized *Escherichia coli*. Lastly, in vivo B-cell responses were assessed by measuring the serum Ig response to SARS-CoV-2 vaccination.

## Methods

### Experimental design

A total of 36 individuals from the 100 plus study cohort, as previously described by Holstege et al [22], were selected for this study (METC number: 2016.440, approved by the Medical Ethics Committee of the VU University Medical Center, Amsterdam, the Netherlands). In short, volunteers either had to be ≥100 years, and self-reported as cognitively healthy, which was confirmed by a family member of close relation [22], or to be the offspring or a sibling of such individuals. When including offspring, the parent had to be a confirmed p.P522R-carrier. After inclusion, peripheral blood (PB) was collected at one or multiple occasions in K3EDTA collection tubes (PB-EDTA), sodium heparin collection tubes (PB-HEP), and serum collection tubes (BD Vacutainer, BD Biosciences, San Jose, Ca, USA). To avoid influence of circadian rhythm on cell counts, samples were always collected in the morning. p.P522R-carriership was determined by Sanger sequencing or Illumina Genome Screening Array (GSA, GSAsharedCUSTOM_20018389_A2, v1, human genome build 37) as described elsewhere [23]. In the current study, we investigated quantities, immunophenotypes, and functions of circulating immune cells in three overlapping cohorts (Cohort I, II and III), described in Table 1. Immunophenotyping was performed on all individuals included in Cohort I (*n* = 33). Calcium flux measurements were performed on cells from donors included in Cohort I and II, with exception of two donors that showed B-cell aberrancies (*n* = 33). Functional evaluation (phosphorylation of PLCγ2, KREC analysis, phagocytosis assay and ROS production) was performed on Cohort II (*n* = 14). Lastly, vaccination responses were evaluated in Cohort III (*n* = 22). Family composition can be found in Table S1. In order to keep the impact of genetic diversity limited, we compared p.P552R effects between siblings whenever possible. Due to more stringent inclusion criteria (based on current health status, known co-morbidities, and medication use) for Cohort II, the effect of p.P522R could only be assessed in one family. The remainder of cohort II was age- and sex-matched.

**Table 1:**
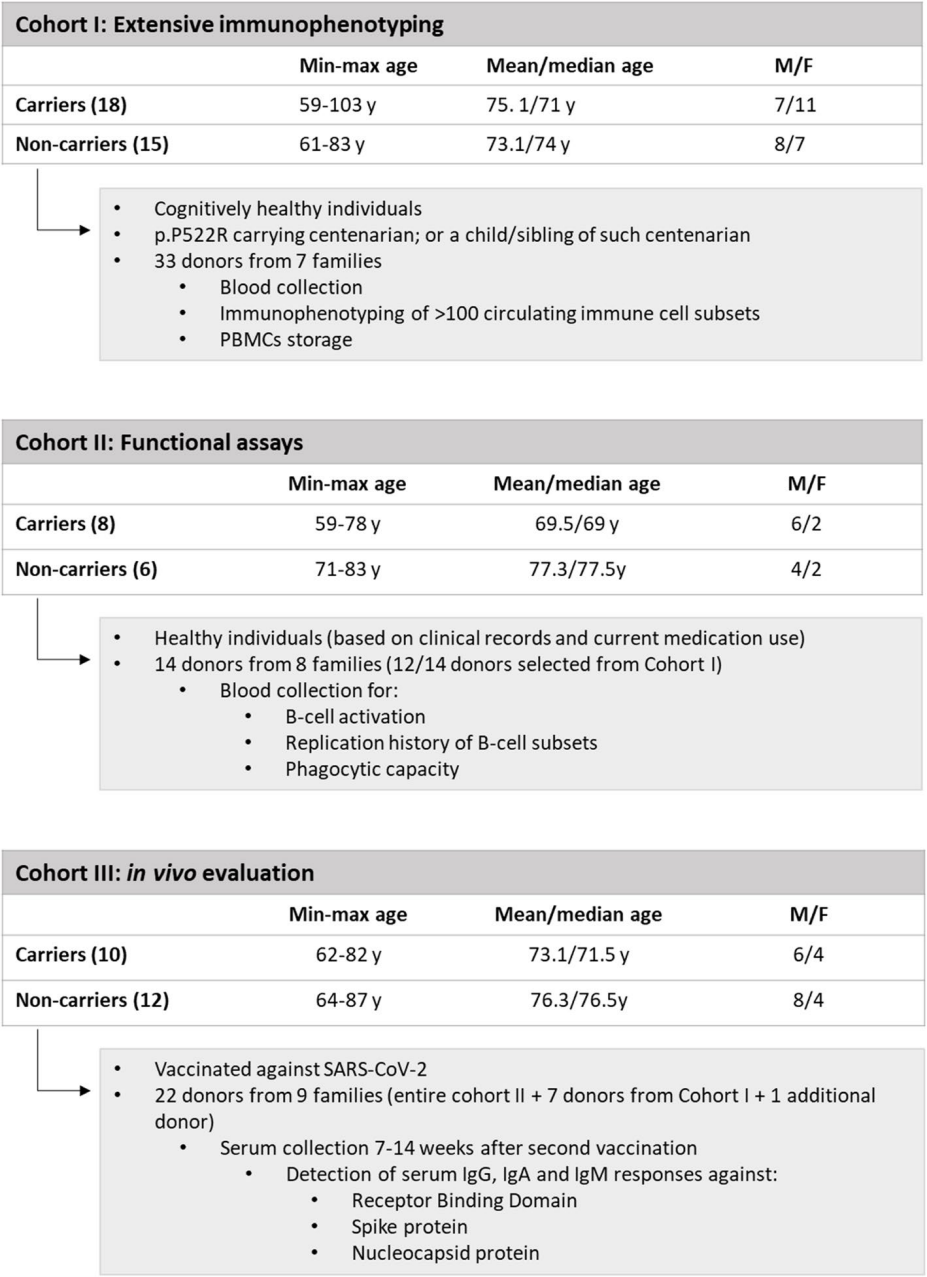
Description of the cohorts used in this study; Cohort I, II, III

*Abbreviations*: Carrier Carrier of *PLCG2* p.P522R, *Non-carrier* Not a carrier of *PLCG2.* p.P522R. *PBMCs* Peripheral blood mononuclear cells. *Ig* Immunoglobulin

### Settings of flow cytometers

For all flow cytometers (available at the Flow cytometry Core Facility at LUMC) there was a daily quality control (QC). QC for Cytek Aurora flow cytometers (Cytek Biosciences, Fremont, Ca, USA) was performed using SpectroFlo QC Beads (Cytek Biosciences) as recommended by the manufacturer. BD FACS LSR Fortessa 4 L and BD FACS Canto II 3 L (BD Biosciences, San Jose, CA, USA) were calibrated and compensated according to EuroFlow guidelines and daily QC was performed using BD™ Cytometer Setup and Tracking (CS&T) beads (BD Biosciences) and Perfect-Count Microspheres™ (Cytognos, Spain), as described before [24, 25].

### Immunophenotyping of circulating immune cells

PB-EDTA from Cohort I was used < 12 h after collection for immunophenotyping. In-depth analysis of circulating innate and adaptive immune cells was performed using previously published flow cytometry panels, or their direct prototypes, and gating strategies (Table S2). Phenotypic descriptions of each population are presented in Tables S3, S4, S5 and S6.

The dendritic cell-monocyte (DC-monocyte) panel allows identification of up to 19 different (sub) populations in the myeloid compartment [26, 27]. The CD4-T cell panel (CD4T) allows identification of at least 89 (sub) populations within the CD4 T-cell compartment, which comprise of different functionalities and maturation stages [27, 28]. The CD8 cytotoxic T-cell (CYTOX) panel allows identification of up to 50 (sub) populations within the CD8 T cells, TCRγδ T-cells and the natural killer (NK) cell compartments [27]. Lastly, the B-cell and plasma cell (BIGH) panel allows identification of up to 115 populations of B and plasma cells, distinguished based on their maturation stage-associated phenotype and the expressed Ig subclasses [27, 29, 30].

Depending on the antibody combination, samples were either processed according to the bulk lysis protocol for staining of 10 × 10^6^ cells (DC-monocyte and BIGH) or prepared using the EuroFlow stain-lyse-wash protocol (CD4T, CYTOX); both protocols available on www.EuroFlow.org. For BIGH and CYTOX tubes, surface staining was followed by intracellular staining with the Fix & Perm reagent kit (Nordic MUbio, Susteren, The Netherlands) according to manufacturer’s protocol. In brief, 100 μL of washed sample was fixed with 100 μL of Solution A (15 min in the dark at RT), washed, and permeabilized by adding 100 μL of Solution B (15 min in the dark at RT) and antibodies against intracellular markers.

After washing, cells were re-suspended in PBS for immediate acquisition (or stored for max ~ 3 h at 4 °C).

An additional flowcytometry panel was used to evaluate expression of non-phosphorylated PLCγ2 in various cell populations (Table S2). Here, samples were processed according to the bulk lysis protocol for staining of 2.5 × 10^6^ cells (www.EuroFlow.org).

For precise enumeration of cells, we used Perfect-Count Microspheres™ (Cytognos) according to the EuroFlow SOP (protocol available on www.EuroFlow.org). In short, 50 μL of well-mixed Perfect Count Microspheres were added to 50 μL of peripheral blood. This mixture was incubated for 30 min with antibodies directed against CD19, CD3 and CD45. Next, 500 μL of NH_4_Cl was added to lyse the red blood cells. After a 10 min incubation with NH_4_Cl, samples were immediately acquired on a BD FACS LSR Fortessa 4 L, or a BD FACS LSR Fortessa X-20 4 L (both BD Biosciences, San Jose, CA, USA).

### Comparison of immune-related SNPs and calculation of polygenic risk score of immune-related SNPs between carriers and non-carriers

To investigate whether other immune-related genetic differences may explain the observed differences between the carriers and non-carriers, we calculated an immune-AD polygenic risk score (PRS). We included 59 SNPs (including the CD33 SNP) that were identified by a recent GWAS [31] and were predicted to have an effect on immune-function by snpXplorer (http://snpxplorer.net/, Table S7 [32]). We excluded the rs72824905 SNP in *PLCG2*. PRSs were compared between p.P522R *PLCG2* carriers and non-carriers. We used logistics regression models to compare AD-PRS and immune-PRS between cases (p.P522R *PLCG2*-carriers) and controls (*PLCG2* wild-type). For the single-SNP association, we used logistic regression models using SNP dosages, and associations were corrected with a False Discovery Rate (FDR < 0.1).

### Measurement of phosphorylated PLCγ2

For in vitro stimulation assays, PB-HEP from Cohort II was used < 12 h after collection. Intracellular expression of phosphorylated PLCγ2 (pPLCγ2) was measured to assess B-cell activation upon stimulation with IgM or IgG Fabs (being F (ab)2 fragment Goat anti-human IgM Heavy Chain secondary antibody (Southern Biotech), and F (ab)2 fragment Goat anti-human IgG (Jackson ImmunoResearch), respectively). First, PB-HEP was subjected to a bulk lysis to remove red blood cells (protocol at www.EuroFlow.org). Then, cells were incubated with an antibody cocktail for surface staining (45 min, RT in the dark) (Table S2), washed with PBS, incubated with 1 μL 1:10 Zombie NIR™ Fixable Viability Dye (BioLegend) for 30 min at RT and washed again. Subsequently, cells were resuspended in 230 μL PBS + 0.5% BSA and incubated with 10 μg anti-IgM F (ab)2 or anti-IgG F(ab)2 fragments (10 min at 37 °C in a shaking water bath). The reaction was stopped by adding 62.5 μL Inside fix (Cell signaling buffer set A; Miltenyi) and incubating for 10 min in the dark. Afterwards, cells were washed and permeabilized (Cell signaling buffer set A, Miltenyi), washed again and resuspended in PBS + 0.5% BSA. Next, antibodies to detect pPLCγ2 and immunoglobulins were added (30 min at RT in the dark). After a final washing step, cells were resuspended in PBS and acquired on a BD FACS Canto II 3 L. Integrated MFI (iMFI) was calculated according to the following Eq. (E1) [33]:

1 $$integrated\ MFI=\% pPLC\gamma 2\ positive\ cells\times MFI\ pPLC\gamma 2\ positive\ cells$$

As the stimulated B-cell receptor could not be targeted for antibody stain to identify cells, the following marker combinations were used to identify B-cell subsets: For IgM stimulation: pre-GC B cells, CD20+CD27-IgG-IgA-; unswitched MBCs, CD20+CD27+IgG-IgA-; class-switched MBCs, CD20+CD27+IgG+ or CD20+CD27+ IgA+. For IgG stimulation: pre-GC B cells, CD20+CD27-IgD+IgA-; unswitched MBCs, CD20+CD27+IgD+IgA-; class-switched IgG MBCs, CD20+CD27+IgD-IgA-.

### Calcium flux assay

Peripheral blood mononuclear cells (PBMCs) from Cohort I and II were isolated from fresh PB-HEP by means of a density gradient (in-house; Ficoll-amidotrizoate, density 1.077 g/mL) and stored in liquid nitrogen (freeze medium; RPMI + 40% FCS + 10% DMSO). At the day of analysis, PBMCs were thawed and washed twice with loading buffer (HBSS + 10 mM HEPES + 5%  FCS). Next, cells (10 × 10^6^ PBMCs/mL) were loaded with Indo-1 Calcium Sensor Dye (Fisher Scientific, final concentration 2 μg/mL) for 30 min at 37 °C, labeled with antibodies directed against cell surface markers and incubated for additional 15 min at 37 °C (Table S2). Subsequently, cells were washed with 10-20x labeling volume in Flux buffer (HBSS + 10 mM HEPES + 5% FCS + 1 mM CaCl_2_) and resuspended in 500 μL Flux buffer. Samples were measured immediately on a Cytek Aurora 5 L flow cytometer.

After establishing a ~ 2.5 min baseline, cells were stimulated with 20 μL (10 μg) anti-IgM F(ab)2 Fragments (Southern Biotech) or 8.3 μL (10 μg) anti-IgG F(ab)2 Fragments (Jackson ImmunoResearch) and measured for ~ 10 min. Finally, ionomycin was added (5 μL, 1 mg/mL in DMSO) and samples were measured for ~ 2.5 minutes. Acquisition was performed at medium speed (~ 30 μL/min). During stimulation and measurement, samples were kept at 37 °C in a tube heating device. Indo-Free and Indo-Bound signal was detected in the UV7 and UV1 detector, respectively. Samples were analyzed by dividing the sample into 30 time slots (of equal time, ~ 30 sec) with the Infinicyt Software (Cytognos) and determining the UV1/UV7 ratios in each time slot. Time slots 1–5 represent baseline signal, time slots 6–25 represent Fab-stimulated signal, and time slots 26–30 represent ionomycin-stimulated signal. MFIs of all time slots were used to plot a curve per B-cell population, from which the area under the curve (AUC) was determined for each individual sample using the GraphPad PRISM Software (v8.1.1). To calculate AUC for the ‘total Fab stimulation’, time slots 6–25 were used. When calculating the AUC for the ‘peak of Fab stimulation’, time slots 7–12 were used, as these were the highest values in all donors and presented the peak of calcium release. ‘Ionomycin stim’ AUC was calculated using time slots 26–30. In all cases, AUC was only calculated for points higher than baseline signal (unstimulated sample). As the stimulated B-cell receptor could not be targeted for antibody stain to identify cells, the following marker combinations were used to identify B-cell subsets: For IgM stimulation: pre-GC B cells, CD20+CD27-IgG-IgA-; unswitched MBCs, CD20+CD27+IgG-IgA-; class-switched MBCs, CD20+CD27+IgG+ or IgA+; and for IgG stimulation: pre-GC B cells, CD20+CD27-IgD+IgA-; unswitched MBCS, CD20+CD27+IgD+IgA-; class-switched IgG MBCs, CD20+CD27+IgD-IgA-.

### KREC analysis to determine cell proliferation history

PB-HEP from Cohort II was used < 24 h after collection for high-speed cell sorting of pre-GC B cells (CD19+CD27-IgM+IgG-IgA-), unswitched MBCs (CD19+CD27+IgM+IgG-IgA-) and class-switched MBCs (CD19+CD27+IgM-IgG+ or CD19+CD27+IgM-IgA+) using a BD FACS Aria III 4 L (BD Biosciences, San Jose, CA, USA). On average, a purity of 98% was reached for pre-GC B cells, unswitched MBCs, and class-switched MBCs. KREC numbers were determined as previously described [34, 35]. In short, DNA of sorted populations and the KREC control cell line (DB01) was isolated with a QIAmp DNA Micro Kit (QIAGEN) and DNA concentrations were determined by NanoDrop 2000 (Thermo Fisher Scientific). Next, we performed qPCR (Quantstudio qPCR Machine, Thermo Fisher Scientific) to quantify the average amount of coding joints (Cj), signal joints (Sj) and Albumin (Alb) in each B-cell population and the control cell line (DB01), using the primers that were previously described by van Zelm and colleagues [34]. The number of cell divisions (ΔCt) for each B-cell population was calculated with the following Eq. (E2):

2 $$\varDelta Ct= Ct(Sj)- Ct(Cj)-\varDelta Ct(control)$$

Where ΔCt (control) is a standard correction for primer efficacy based on the Ct(sj) and Ct(Cj) from DNA from the DB01 control cell line.

### Phagocytosis of pHRodo™ Green *E. coli* bioparticles

Assessment of the phagocytic capacity was performed with opsonized *E. coli* (pHRodo™ Green *E. coli* Bioparticles, ThermoFisher). Polystyrene FACS tubes (4 mL) were filled with 200 μl PB-HEP (Cohort II, < 12 h after collection) and placed on ice for at least 10 minutes. Then, 40 μL of pHRodo™ Green *E. coli* was added, samples were mixed and incubated at 37 °C for exactly 20 min. All further steps were performed on ice or at 4 °C to stop the reaction. Cells were washed and stained (30 min on ice in the dark) with an antibody cocktail (Table S2). Next, samples were lysed with BD lyse (10 min, rolling at 4 °C). Lastly, cells were washed, resuspended in cold PBS with 0.5% BSA and acquired at a Cytek Aurora 3 L flow cytometer. To prevent shedding of CD62L, TAPI-2 (final concentration of 20 μM) was always present. To account for background activation or signal, several control tubes were measured in addition to the sample tubes (Table S8).

### Production of reactive oxygen species (ROS)

Production of ROS upon stimulation was assessed with the phagoBURST kit (PHAGOBURST™ CE/IVD kit, BD Biosciences) according to manufacturer’s protocol with some modifications, as described further in this paragraph. In short, each tube was filled with 200 μL PB-HEP (Cohort II, < 12 h after collection) and placed on ice for at least 10 minutes. Then, 20 μL of wash buffer (reagent A), Phorbol 12-Myristate 13-acetate (PMA) (Sigma, P8139-1 mg, final concentration 600 ng/mL), or *E. coli* (reagent B, well-mixed by pipetting) was added to each tube. After proper mixing, tubes were incubated for exactly 10 min at 37 °C in a shaking water bath. Next, 20 μL of substrate (dihydrorhodamine -DHR123, reagent E) was added to each tube and mixed. Tubes were incubated for another 10 min at 37 °C. Subsequently, the assay was stopped by lysing the cells (reagent F, 15 min, RT, dark). Cells were washed twice, stained for 15 min in the dark at RT with antibody cocktail (Table S2) and PBS with 0.5% BSA, washed again, resuspended in PBS with 0.5% BSA and stored on ice until acquisition on a Cytek Aurora 3 L flow cytometer (< 1 h). To prevent shedding of CD62L, TAPI-2 (final concentration of 20 μM) was always present. The rhodamine 123 (R123) signal was used as a readout for ROS production (conversion from DHR123 to R123). To account for background activation or signal, several control tubes were measured in addition to the sample tubes (Table S9).

### Data integration phagocytosis and ROS production

The phagocytosis assay and the ROS production were considered complementary assays in which we evaluated three aspects of phagocytosis. First, we evaluated the percentage of phagocytosing cells, then we combined this – together with the pHRodo™ Green signal- into the integrated MFI (iMFI) [33], which was used as a measure of how many particles each cell has phagocytosed (E3). For ease of interpretation, this value was divided by 100,000.

3 $$integrated\ MFI=\%E.\ coli\ positive\ cells\times MFI\ E.\ coli\ positive\ cells$$

Lastly, we measured the production of ROS upon phagocytosis and combined this with the iMFI into one output; the normalized ROS production, which was defined as the ROS generation per given number of phagocytosed particles (E4).

4 $$normalized\ ROS\ production=\frac{R123\ MFI}{integrated\ MFI}$$

### In vivo evaluation

We collected additional blood samples from donors (Cohort III), 7–14 weeks after their second vaccination against SARS-CoV-2. Serum antibodies directed against the Spike (S) protein, Receptor Binding Domain (RBD) and Nucleocapsid (N) protein were determined by a fluorescent-bead-based multiplex immunoassay (MIA), as previously described [36]. In short, the stabilized pre-fusion conformation of the ectodomain of the Spike protein, the Receptor Binding Domain of the S-protein (RBD) and the Nucleocapsid (N) protein were each coupled to beads or microspheres with distinct fluorescence excitation and emission spectra. Serum samples were diluted and incubated with the antigen-coupled microspheres. Following incubation, the microspheres were washed and incubated with phycoerythrin-conjugated goat anti-human, IgG, IgA, and IgM. The data were acquired on the Luminex FlexMap3D System and MFI was converted to international units per milliliter (IU/mL), using Bioplex Manager 6.2 (Bio-Rad Laboratories) software.

### Data analysis and statistical analysis

Flow cytometry data was analyzed with Infinicyt software (version 2.0.3.a. and 2.0.4.b., Cytognos, Spain). Statistical analysis was performed in GraphPad Prism 8.1.1 software (GraphPad, San Diego, CA, USA). Differences between p.P522R-carriers and non-carriers, were evaluated using the Mann-Whitney U test. Impact of age was assessed using Spearman’s correlation. *P* < 0.05 was considered significant.

## Results

### Cell count analysis and immunophenotyping (cohort I)

#### Numbers of circulating immune cells in the study cohort are representative for healthy aged adults

The median age of the individuals studied in Cohort I was 72 years (range: 59 to 103 years); one donor was a sibling, and 30 were children of a centenarian p.P522R variant-carrier, and two were centenarians (details provided in Methods section and Table 1). To reduce the possible impact of sex or age on the comparison between p.P522R-carriers and non-carriers, Cohort I was sex- and age-matched as much as possible (this was also true for Cohorts II and III, described below).

We first compared absolute counts of multiple innate and adaptive immune cell populations as observed in Cohort I with reference values from healthy age-matched cohorts [26, 28, 29]. Overall, innate, CD4 T- and B-cell counts in the donors were representative of their age category, with exception of IgG3, IgG4 and IgA2 plasma cells, which were slightly elevated in several donors (data not shown). For CD8 T- and NK-cell subsets no age-matched reference values were available. We observed no association with sex (data not shown) but we observed a pronounced effect of pedigree, as in several subsets cell counts from all first-degree family members tended to cluster together, even when individuals were living at different locations for many years (Fig. S1). Of note: we observed a subclinical B-cell expansion in three donors from one family, all p.P522R-carriers. Additionally, after initial screening, two donors (non-carriers from different families) had expanded B-cell populations with phenotypes CD19+CD20+CD5+CD21+CD27+IgM+IgD−/dim and CD19+CD20+CD5+CD21dimCD27+IgG1dim); these individuals were referred to a hemato-oncologist for further evaluations and excluded from downstream analyses.

#### Differences in immunophenotype between p.P522R-carriers and non-carriers

**Fig. 1:**
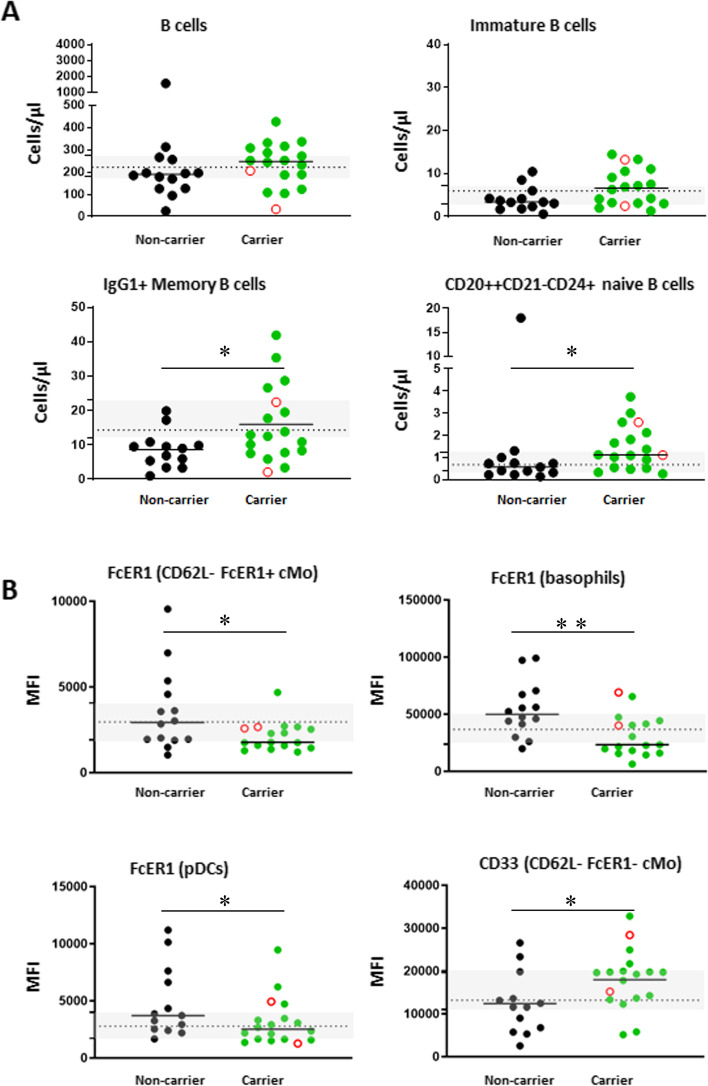
Main differences between PLCγ2 p.P522R-carriers and non-carriers in Cohort I. **A** Major differences in absolute immune cell counts between carriers and non-carriers. **B** Major differences in activation marker expression between carriers and non-carriers. Mean fluorescence intensity (MFI) was corrected by subtraction of background signal on a negative reference population. Differences between cohorts were determined using the Mann-Whitney U test. Green circles indicate p.P522R-carriers, and black circles indicate non-carriers. Centenarian data points are indicated in the graphs as an open red circle. Grey boxes indicate the 95%CI and dashed lines indicate the median cell count or MFI from a younger reference cohort (*n* = 25, average age: 31 years old), whose data was collected in the same laboratory, using identical methods and equipment. *N* = 31, * *p* < 0.05, ** *p* < 0.01

We evaluated the impact of PLCγ2 carriership on cell counts in innate and adaptive immune cell subsets. We observed the largest differences in cell counts between carriers and non-carriers in total B cells, immature B cells, CD20++CD21-CD24+ naive B cells (0.56 cells/μL in non-carriers vs 1.12 cells/μL in carriers, *p* = 0.0191) and IgG1+ MBCs (8.98 cells/μL in non-carriers vs 12.73 cells/μL in carriers, *p* = 0.0420) of which the last two reached statistical significance (Fig. 1A). This pattern was observed in at least half of the families with mixed carriership (CD20++CD21-CD24+ naive B cells; 3/6, IgG1+ MBCs; 5/6 families). Interestingly, upon comparing median cell counts in carriers and non-carriers with a reference cohort comprising counts from 25 individuals (aged 18–54, median age: 27 years, 9 females) we observed that non-carriers had lower IgG1+ MBC counts compared to the p.P522R**-**carriers or the younger reference cohort (p = 0.0420 and *p* = 0.0009, respectively) (Fig. 1A). For total B cells, immature B cells, and CD20++CD21-CD24+ B cells, this difference did not reach statistical significance.

Next, we evaluated expression of several surface markers: HLA-DR, CD62L, CD16, CD33 and FcεRI on innate immune cells, CD45RA, CD27, CD28 and CD3 on T cells, and CD20 and CD21 on B cells. Interestingly, we found a reduced expression of FcεRI on CD62L-FcεRI+ classical monocytes (CD62L-FcεRI+ cMo, MFI 2929 vs 1771, *p* = 0.0397), basophils (MFI 49861 vs 23,288, *p* = 0.0040) and plasmacytoid dendritic cells (pDCs, MFI 3706 vs 2513, *p* = 0.0464) in p.P522R-carriers versus non-carriers (Fig. 1B). Moreover, the expression of CD33 on CD62L-FcεRI- cMos was increased in p.P522R carriers (MFI 11599 vs 19,407, *p* = 0.0342). We observed no statistically significant differences compared to the younger reference cohort.

Notably, differences between p.P522R-carriers and non-carriers were unlikely to be influenced by other immune-related genetic risk-factors associated with AD. We generated a polygenic risk score (PRS) comprised of all genetic variants that associated with the immune system, as currently identified in the AD-GWAS (Table S7) [31]. A comparison of the AD-immune PRS between p.P522R-carriers and non-carriers in Cohort I indicated that, aside from the *PLCG2* protective variant, there were no other immune-related genetic differences. Also, single variants occurred with similar frequencies in carriers vs non-carriers.

#### Higher PLCγ2 expression in immune cells of p.P522R-carriers

**Fig. 2:**
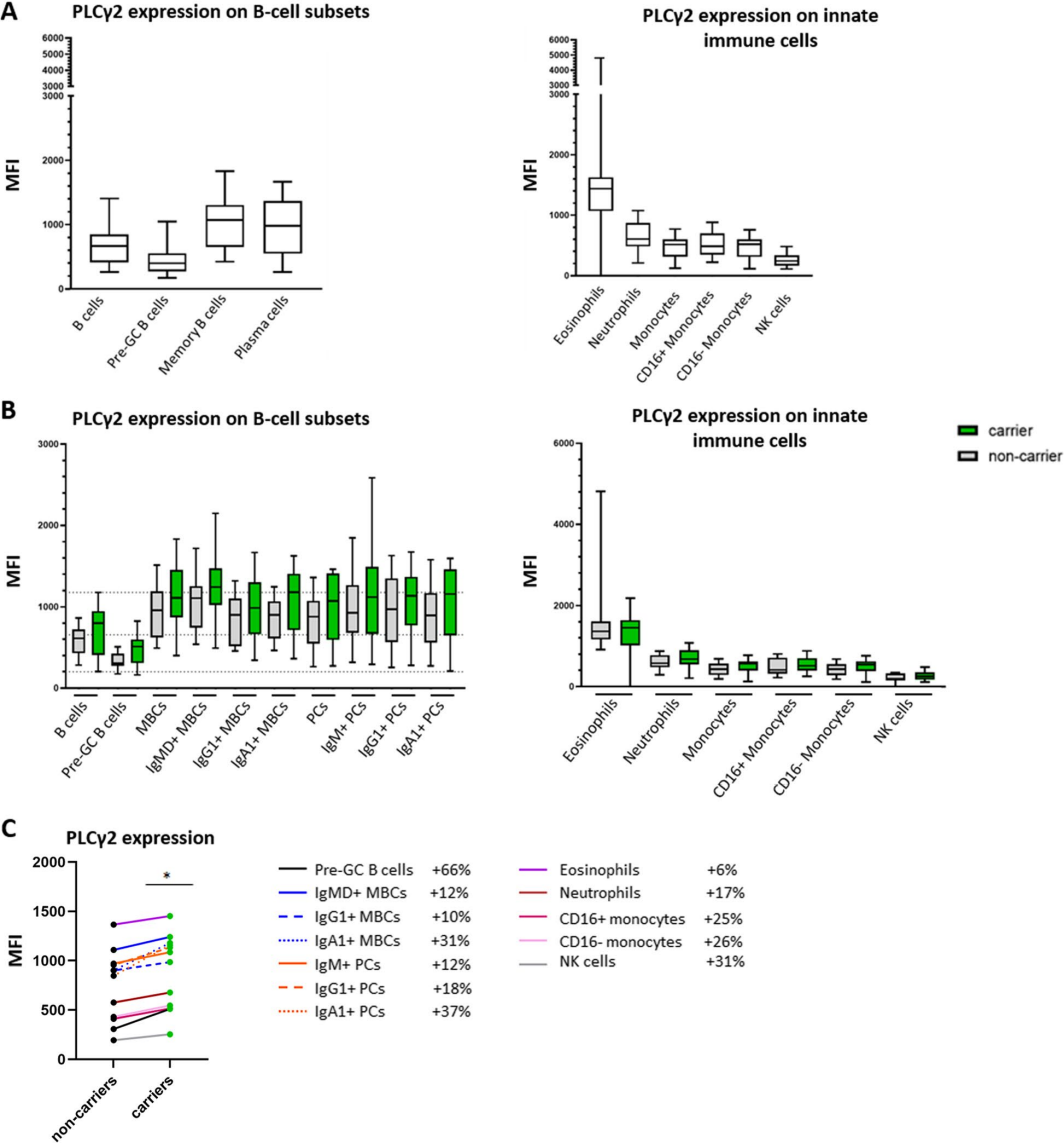
PLCγ2 expression in different populations of circulating immune cells. **A** PLCγ2 expression measured as mean fluorescence intensity (MFI) and corrected for background signal by subtracting the MFI signal of a negative reference population (T cells) of the total cohort (irrespective of p.P522R-carriership). *N* = 31. **B** PLCγ2 expression on B-cell and myeloid cell subsets in p.P522R-carriers (green) and non-carriers (grey). Dashed lines represent the median (min-max) of PLCγ2 expression of B cells of the total cohort. **C**PLCγ2 expression on B-cell and myeloid cell subsets between carriers (green) and non-carriers (black). The median of each subset is plotted, medians of the same subset are connected with lines. % increase in PLC**γ**2 expression in carriers vs non-carriers is indicated behind each population in the legend. Differences were evaluated with Mann-Whitney U test. MBC; memory B cell, IgMD+ MBC; unswitched memory B cell, Pre-GC; pre-Germinal Center B cells, PC; plasma cells, NK cells; Natural Killer cells. * *p* < 0.05

Next, we evaluated the PLCγ2 expression levels in different leukocyte populations in Cohort I (irrespective of p.P522R**-**carriership). We observed the highest PLCγ2 expression levels in eosinophils, albeit with high inter-individual variability, and in antigen-experienced B cells which was consistently high across all individuals (Fig. 2A). Interestingly, the median levels of PLCγ2 expression in all evaluated B-cell subsets were consistently increased in p.P522R-carriers than in non-carriers (range + 10 to + 66%) (Fig. 2 BC). In innate immune cells, median PLCγ2 expression levels were only slightly elevated in p.P522R-carriers compared to non-carriers (range from + 6 to + 31%) (Fig. 2 BC).

To summarize our findings based on cell counts: Cohort I was representative of healthy individuals of this age group. We observed several differences in cell counts and expression of activation markers between p.P522R-carriers and non-carriers. PLCγ2 expression tended to be higher in carriers, mostly on antigen-experienced B cells. p.P522R-carriers and non-carriers had similar AD-immune PRS scores, such that differences were unlikely to be influenced by other immune related genetic factors associated with AD.

### Functional B-cell analysis (cohort II); response to SARS-CoV-2 vaccination (cohort III)

#### Higher levels of phosphorylated PLCγ2 in stimulated B cells from p.P522R-carriers

**Fig. 3:**
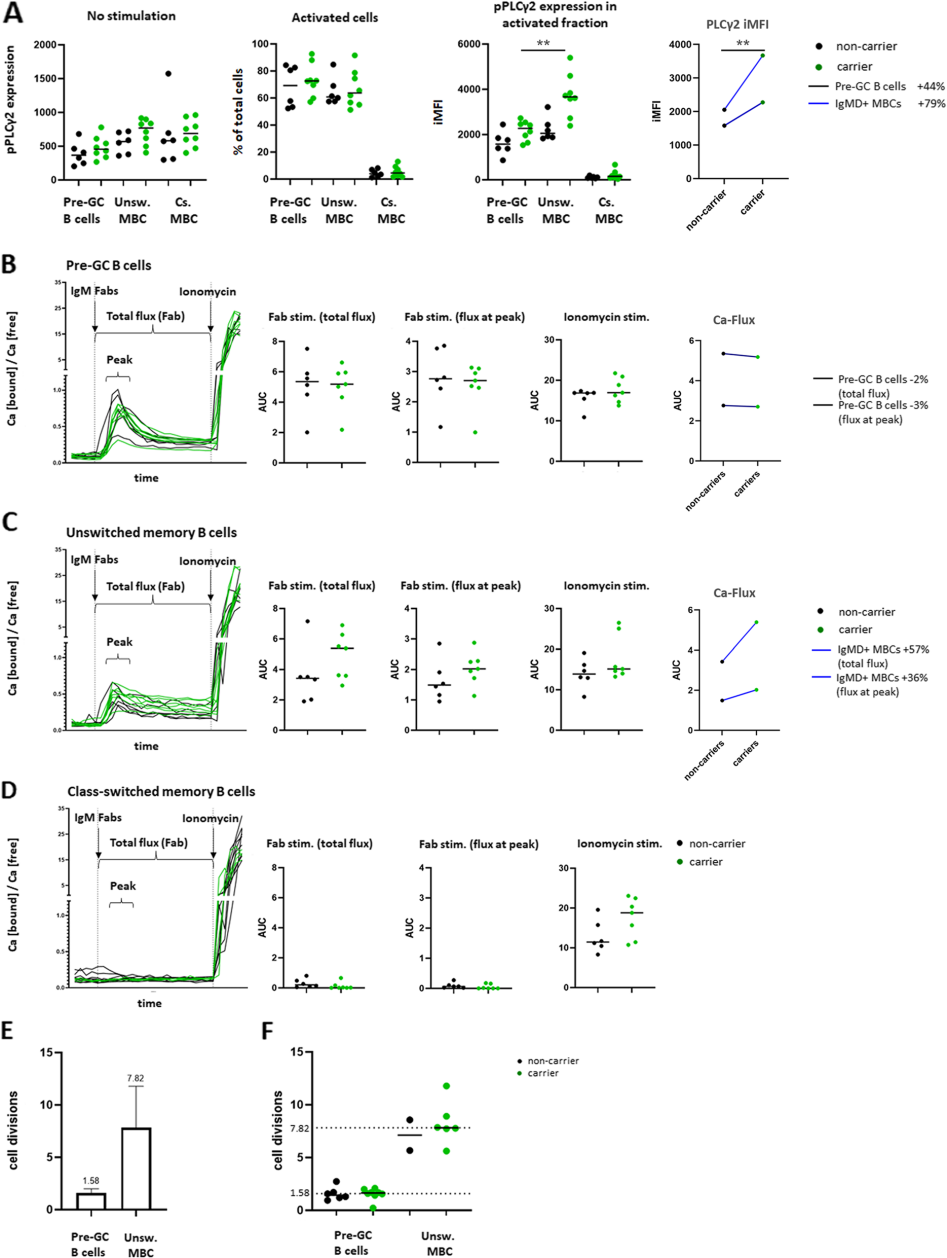
Assessment of B-cell activation upon IgM Fab stimulation and B-cell replication history in p.P522R-carriers and non-carriers. **A** Expression of phosphorylated PLCγ2 (pPLCγ2) in B cells before and after stimulation of the B-cell receptor with IgM Fabs. Detection of pPLCγ2 was used as a direct measure of B-cell receptor activation after IgM Fab stimulation. *N* = 14**.** % increase in PLC**γ**2 phosphorylation in carriers vs non-carriers is indicated behind each population in the legend. **B-D** Measurement of calcium release (‘flux’) after B-cell stimulation with IgM Fabs of pre-GC B cells (CD27-IgG-IgA-) **B,** unswitched memory B cells (CD27+IgG-IgA-) **C**, or class-switched memory B cells (CD27+IgG+ or CD27+IgA+) **D** in cohort II. Differences between carriers and non-carriers were evaluated by comparing the area under the curve (AUC) of the total Fab stimulation (from stimulation until the moment ionomycin was added, ~ 10 min, flux intensity and duration), the peak of the response after Fab stimulation (the 5 highest points after the Fabs were added to the cells; flux intensity), and after ionomycin was added (to determine the maximum flux). AUC was calculated only for points that were higher than baseline value (unstimulated sample). *N* = 13 (one sample was lost due to technical failure). % increase or decrease in AUC in carriers vs non-carriers is indicated behind each population in the legend. **E + F** Measurement of the average number of undergone cell divisions by total pre-GC B cells (CD27-IgM + IgG-IgA-) or unswitched memory B cells (CD27+IgM+IgG-IgA-) by means of qPCR-based KREC assay in the total cohort **E** or separated into carriers and non-carriers **F**. For pre-GC B cells; *n* = 14. Due to low DNA concentration, for unswitched MBC; *n* = 8. Differences were evaluated by Mann-Whitney U test. Pre-GC; pre-Germinal Center, MBC; memory B cells, iMFI; integrated mean fluorescence intensity (for calculation, see Methods) Unsw. MBC; unswitched memory B cells, Cs. MBC; class-switched memory B cells. ** *p* < 0.01

In a second analysis, we evaluated the effect of p.P522R on PLCγ2 activity in several B-cell subsets derived from Cohort II, comprising 14 healthy older adults, aged 59–83, of whom 8 p.P522R-carriers and 6 were WT (Table 1, Text S1). p.P522R-carriers and non-carriers in Cohort II had a similar AD-immune polygenic risk score, and single variants occurred with similar frequencies in both groups, suggesting that carriership of other genes are unlikely to influence findings. As PLCγ2 is located downstream of the BCR, we assessed whether specific elements of the signaling pathway were affected by the carriership status. The BCR was stimulated with IgM (stimulation with IgG Fabs was also tested, but due to high background signal, results were considered unreliable). The PLCγ2 APC antibody used in this study recognizes activated PLCγ2 (pPLCγ2) after phosphorylation at tyrosine 759 (pY759), i.e. one of the two phosphorylation sites (Y759 and Y753). In the steady state, we observed no significant difference between the levels of pPLCγ2 in B cells from p.P522R-carriers and non-carriers (Fig. 3A). Upon BCR stimulation with IgM Fabs, a similar percentage of unswitched MBCs was activated in p.P522R-carriers and non-carriers, but the integrated mean fluorescent intensity (iMFI) of PLCγ2 was 79% higher in carriers than in non-carriers (iMFI 3667 vs 2051, respectively, *p* = 0.0047), implying a stronger activation in carriers (Fig. 3A). Although the increase in pPLCγ2 was only significant for unswitched memory B cells, we observed that in pre-GC B cells the levels of pPLCγ2 after stimulation also tended to be higher in carriers (pre-GC B cells + 44%; unswitched MBCs + 79%). According to expectations, no increase in pPLCγ2 was observed upon IgM stimulation in the (IgM-) class-switched MBCs in either group.

#### Higher calcium flux in B cells upon BCR stimulation in p.P522R-carriers

Next, we evaluated the effect of p.P522R further downstream of the BCR, by measuring the calcium release (‘calcium flux’) upon BCR stimulation using IgM Fabs. After a pilot experiment using IgM and IgG Fab stimulation in 12 donors, we found IgM stimulation to be most robust (Fig. S2). We observed no differences between p.P522R carriers and non-carriers in calcium flux in pre-GC B cells derived from Cohort II donors (‘Total flux’ -2% and ‘ Flux peak’ -3%, Fig. 3B). However, upon Fab stimulation in unswitched MBCs, we observed a trend (not significant) towards higher calcium flux in p.P522R-carriers vs non-carriers (‘Total flux’ + 57% and ‘Flux at peak’ + 36%) (Fig. 3C). This trend was not observed when comparing the calcium flux of all donors included in cohort I and II (Fig. S3). Since stimulation with IgM Fabs did not lead to calcium release in class-switched MBCs, the measured calcium flux is likely due to BCR-specific stimulation (Fig. 3D).

#### No difference in number of cell divisions between p.P522R-carriers and non-carriers

Stronger B-cell activation in p.P522R-carriers upon stimulation may result in more robust proliferation upon antigen encounter. To test this hypothesis, we evaluated B-cell replication history with the KREC (kappa-recombination excision circle) assay. In line with previous publications [35], we found that, across 14/14 individuals, pre-GC B cells had undergone on average 1.58 cell divisions, while across 8/14 individuals in Cohort II, unswitched MBCs had undergone on average 7.82 cell divisions (Fig. 3E). We observed no significant differences in replication history of B-cell subsets between p.P522R-carriers and non-carriers, possibly due to the limited number of donors in the non-carrier cohort (replication history in pre-GC B cells: 1.43 vs 1.64; in unswitched MBCs: 7.12 vs 7.82 (Fig. 3F)). Lastly, although class-switched MBC samples were collected as well, cell numbers were too low to determine the number of cell divisions.

#### Serum Ig responses to SARS-CoV-2 vaccination comparable between p.P522R-carriers and non-carriers

**Fig. 4:**
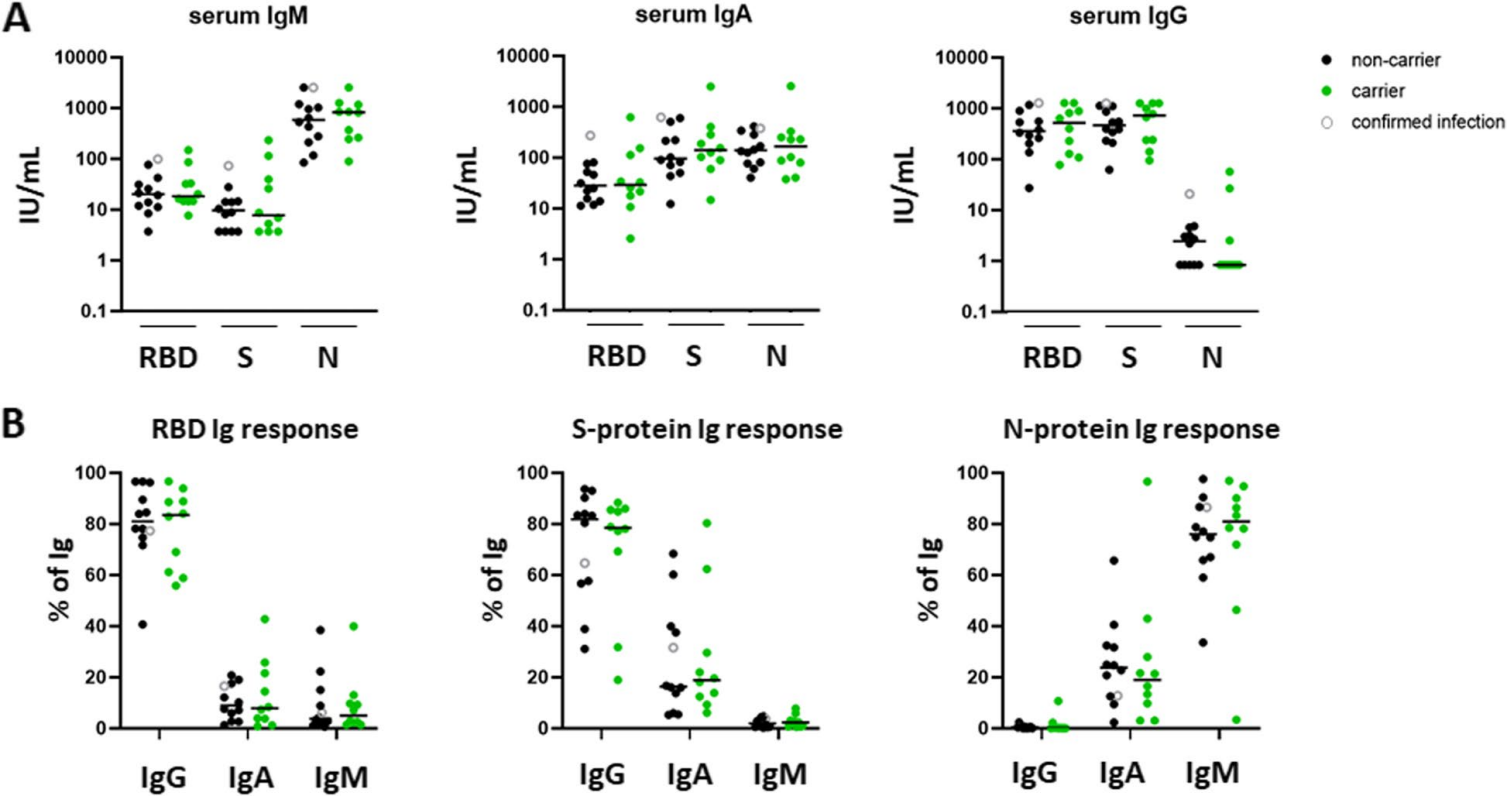
Response to SARS-CoV-2 vaccination in p.P522R-carriers and non-carriers. Samples were donated 7–14 weeks after second vaccination. Type of vaccine received by donors: Comirnaty® (Pfizer/BioNTech) – 9 carriers, 9 non-carriers; Spikevax® (Moderna)- 1 carrier, 0 non-carriers; Vaxzevria® (AstraZeneca) – 0 carriers, 1 non-carrier; vaccine type not indicated- 0 carriers, 2 non-carriers. One donor reported a confirmed SARS-CoV-2 infection, this donor is indicated with an open grey circle. **A** Serum IgM, IgA, and IgG response against the Receptor Binding Domain (RBD), spike protein (S-protein) and nucleocapsid protein (N-protein), expressed in IU/mL. **B** Composition of the serum Ig response per antigen, expressed as % of total antigen-specific Ig response

To test whether the effect of p.P522R on B-cell activation as observed in our in vitro experiments also translates to an in vivo effect (e.g. effect on antibody production), we recognized a window of opportunity in the current global vaccination efforts against SARS-CoV-2. We collected serum samples from 22 individuals (Cohort III), 7–14 weeks after receiving a second vaccination against SARS-CoV-2. Donors were vaccinated with Comirnaty® (Pfizer/BioNTech, 9 carriers, 9 non-carriers); Spikevax® (Moderna, 1 carrier); Vaxzevria® (AstraZeneca, 1 non-carrier), or did not indicate vaccine type (2 non-carriers). All donors developed prominent IgG responses against the viral Receptor Binding Domain (RBD) and Spike (S)-protein. Antibody responses against the viral Nucleocapsid (N)-protein – indicative of viral infection- were predominantly IgM responses (Fig. 4AB). No differences were observed between p.P522R-carriers and non-carriers.

Summarizing our findings in B-cells in Cohort II/III: PLCγ2 in unswitched MBCs is more strongly phosphorylated upon stimulation in p.P522R-carriers compared to non-carriers. Although not statistically significant, the median calcium flux in p.P522R-carriers tended to be increased relative to non-carriers in individuals from Cohort II. Due to technical limitations, we were unable to obtain reliable results for class-switched memory cells, such that it is currently unclear to what extent these data can be extrapolated to other MBC subsets. Despite clear differences between p.P522R-carriers and non-carriers in the multiple in vitro functional experiments, in vivo antibody responses raised against SARS-CoV-2 vaccine were comparable between carriers and non-carriers.

### Innate immune cell analysis (cohort II)

#### Increased ROS production upon FcR-mediated stimulation of innate immune cells in p.P522R-carriers

Besides being located downstream of the BCR, PLCγ2 is also located downstream of FcRs, which are present on many cells of the innate immune system, including the microglia of the brain. Therefore, we evaluated ROS production and phagocytic activity upon FcR-mediated stimulation in several cell types of the innate immune system: neutrophils, classical, intermediate, and non-classical monocytes (cMos, iMos and ncMos, and their subsets), and myeloid and plasmacytoid dendritic cells (mDCs, pDCs, and their subsets).

**Fig. 5:**
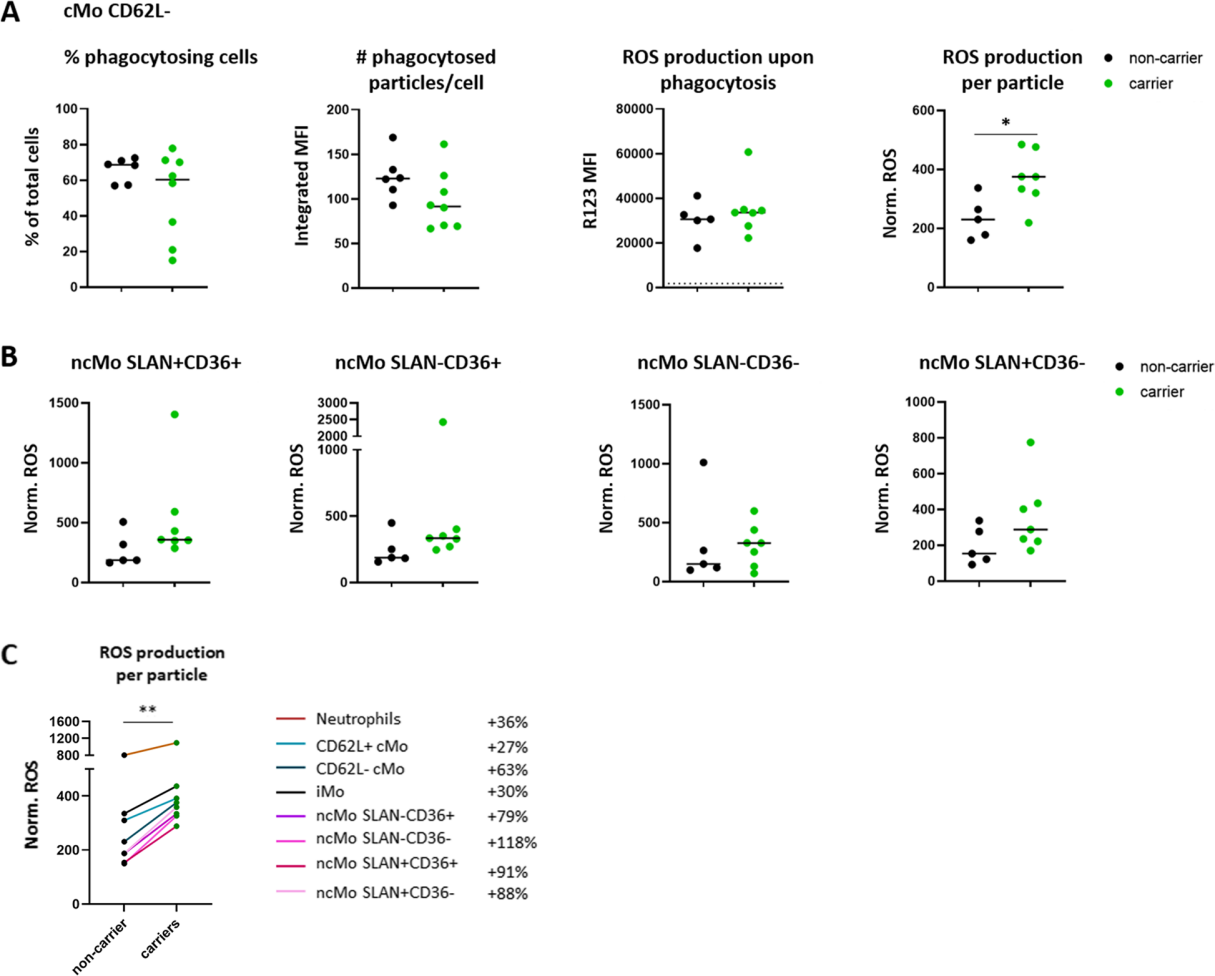
Detection of phagocytosis and ROS production in monocyte subsets after stimulation with pHRodo™ Green *E. coli* bioparticles. **A** To evaluate the outcome of the phagocytosis assays, three different readouts were used per population; % of cells that were phagocytosing, the average amount of particles phagocytosed per cell, and the ROS production upon phagocytosis (as measured by conversion of DHR123 into R123). The average amount of particles phagocytosed per cell and the ROS production were further combined into one value; the ROS produced per particle, or the ‘normalized ROS’ (calculations are indicated in the Methods). The values are presented for the CD62L- classical monocyte (cMo) subset **B** The normalized ROS of all four defined non-classical monocyte (ncMo) subsets, based on expression of CD36 and SLAN. Differences were evaluated by means of Mann-Whitney U test. All readouts were corrected for background/baseline activation by subtracting the value of the control (ice) from the activated (37 °C) sample. * *p* < 0.05. **C** median normalized ROS production in neutrophils and all monocyte subsets. % increase in normalized ROS in carriers vs non-carriers is indicated behind each population in the legend

The percentage of phagocytosing cells did not differ between p.P522R-carriers and non-carriers (Fig. 5A). However, we observed a trend towards lower iMFI in carriers, reflecting a decrease in the number of phagocytosed *E. coli* particles per cell in carriers (phagocytic capacity). This trend was especially observable in classical monocytes (non-carriers vs carriers; cMo CD62L+: iMFI 126.8 vs 109.5, cMo CD62L-: iMFI 122.8 vs 91.58) and non-classical monocyte subsets (non-carriers vs carriers; ncMo SLAN-CD36+: iMFI 105.0 vs 82.31, ncMo SLAN-CD36-: iMFI 72.54 vs 51.75, ncMo SLAN+CD36+: iMFI 72.19 vs 49.74, ncMo SLAN+CD36-: iMFI 50.22 vs 32.02), and less prominent in CD14- mDC subset (non-carriers vs carriers; CD1c CD14- mDCs: iMFI 6.465 vs 3.400) and iMos (non-carriers vs carriers; iMFI 105.4 vs 89.92), and not observed in CD14dim mDCs (non-carriers vs carriers; iMFI 13.24 vs 13.17) (Fig. 5A, Fig. S4).

The level of ROS production upon phagocytosis of opsonized *E. coli* particles (as measured in PhagoBURST assay) was not different between p.P522R-carriers and non-carriers (Fig. 5A, Fig. S4). To combine information from both assays, we investigated the ROS production relative to the number of phagocytosed particles: ‘normalized ROS production’. Normalized ROS production was increased in carriers, which was especially evident in CD62L-cMos (non-carriers vs carriers; ‘normalized ROS’ of 230.4 vs 375.5, *p* = 0.048, Fig. 5A). Although not statistically significant in any other cell population, the median level of ROS production relative to the number of phagocytosed particles tended to be increased in p.P522R-carriers compared to non-carriers and consistently higher across all evaluated monocyte subsets and neutrophils (range: + 27 to + 118%), Fig. 5BC, Fig. S4A-C). Lastly, we observed increased normalized ROS production in CD14- mDCs, but not in CD14dim mDCs (Fig. S4DE). Thus, in several cell populations, the ROS generation per particle tends to be slightly higher in p.P522R-carriers compared to non-carriers.

Additionally, we stimulated samples with Phorbol 12-Myristate 13-acetate (PMA), which results in FcR-independent ROS generation. Surprisingly, we observed a trend towards *decreased* ROS production in p.P522R-carriers compared to non-carriers, which was seen for neutrophils, monocyte subsets and DC subsets (Fig. S5). This effect was the strongest in monocytes, especially in CD62L+ and CD62L- cMos, where p.P522R-carriers showed a significantly lowered ROS production compared to non-carriers.

Thus, upon stimulation with opsonized *E. coli,* we observed that p.P522R-carriers seemed to have a slightly lower phagocytic capacity despite overall similar ROS production upon phagocytosis of *E. coli* and thus had increased ROS production relative to the number of opsonized particles. Nevertheless, the total FcR-independent ROS generation was lower in these same carriers. In general, differences were most prominent in the monocyte subpopulations.

## Discussion

With this work, we present the first study that investigated the effect of the p.P522R variant in PLCγ2 on the human immune system ex vivo and in vivo. We speculated that only subtle changes will be tolerated to translate to the advantageous effect observed in the genetics studies [9, 10], since the molecular interplay that makes up a robust immune system function has evolved under strong evolutionary selection. Thus, it was in full accordance with our expectations that across multiple experiments, in both the B-cell and myeloid cell compartment, the effect of carrying a p.P522R variant was limited. However, all effects pointed in the same direction: an increased sensitivity of the immune cells upon receptor stimulation. Our findings support findings in in vitro cell line and in vivo mouse studies, which indicated that PLCγ2 p.P522R is a gain-of-function variant, exerting a protective effect by slightly increasing PLCγ2 activity [14, 20, 21]. This aligns with recent reports indicating that loss-of-function variant p.M28L, associated with increased AD risk, leads to a reduced PLCγ2 expression in brains [37], suggesting that *reduced* activation of PLCγ2 in PBMCs is a molecular characteristic of AD [38].

### Stronger B cell activation

Our data as measured in blood samples from individuals selected to be least affected by comorbidities or autoimmune disease, suggests that the protective effect of the p.P522R-allele is, at least in part, supported by a stronger B-cell activation in carriers compared to non-carriers. We found that in the steady state the average expression level of PLCγ2 was slightly increased in B cells from p.P522R-carriers compared to non-carriers. Furthermore, upon stimulation of the BCR of unswitched MBCs, the average level of phosphorylated PLCγ2 was significantly increased in p.P522R-carriers compared to non-carriers, and the average calcium flux seemed also slightly increased for unswitched MBCs. Technical limitations prevented confirmation of this pattern in class-switched MBCs, which may be differentially activated [39], such that future studies are required. Also, we acknowledge that we did not observe this signal upon evaluation the Ca-Flux in a more heterogenous cohort of individuals, who potentially have an affected immune response due to diverse comorbidities such as (undiagnosed) autoimmune disease, or use of medication including steroid or non-steroid anti-inflammatory drugs (NSAIDs). Lastly, p.P522R-carriers had higher numbers of circulating CD20++CD21-CD24+ naive B cells and IgG1+ MBCs. Previous studies indicated that with increasing age, the diversity of the B-cell repertoire decreases [40, 41]. The higher number of CD20++CD21-CD24+ naive B cells possibly represents a broader B-cell repertoire allowing carriers to deal with neoantigens more adequately, and may leave the p.P522R-carriers better protected against infections.

To what extent a more sensitive B-cell response has a protective effect on the brain remains to be investigated. However, recent reports suggest that the brain may not be as immune-privileged as previously assumed. B cells have been found to be constitutively present in the dural meninges while small numbers of B-cells may enter the brain parenchyma [42]. Furthermore, (natural) antibodies against brain amyloid or tau have been observed not only in Alzheimer's disease patients, but also in cognitively healthy elderly individuals [43, 44]. In fact, in brain proteinopathies, antibodies can enter the brain parenchyma and effectively remove aberrant proteins, making antibodies promising candidates for intervention strategies for several types of neurodegenerative diseases [45–47]. We thus speculate that a sensitive B-cell system, such as observed in the p.P522R-carriers, through increased activity in the periphery, may contribute to the long-term maintenance of brain health.

### Decreased FcεRI expression on myeloid cells

In myeloid cells, we found that compared to non-carriers, p.P522R-carriers had a lowered expression of FcεRI on pDCs, basophils and CD62L-FcεRI+ cMos. It is known that allergy-related expression of IgE associates with an upregulation of FcεRI expression [48]. Yet the frequency of reported allergy could not explain the decreased FcεRI expression among carriers (in fact, 5/7 reported allergies were in p.P522R-carriers). Upon activation, FcεRI contributes to the production of important immune-mediators, such as cytokines, interleukins, leukotrienes, and prostaglandins, that promote inflammation [49, 50]. Perhaps, this lowered FcεRI expression is an indication of less inflamm-aging in the p.P522R carriers, yet this should be confirmed in a larger study.

### Slightly reduced phagocytosis by myeloid cells of p.P522R carriers

We found that the number of phagocytosed particles per cell tended to be slightly reduced in the myeloid cells of p.P522R-carriers. This aligns with our observation that innate immune cells in p.P522R-carriers had a higher expression of CD33 (up to ~ 1.67-fold in CD62L-FcεRI- cMo) which, upon stimulation by any molecule with sialic acid residues (such as glycoproteins or glycolipids), results in a cascade that inhibits phagocytosis [51]. Notably, previous studies indicated that higher CD33 expression levels, resulting from carrying the common CD33 risk allele (rs3865444) [51, 52] associated with an increased chance of developing Alzheimer’s disease [53–55]. Carriers of this risk allele were reported to have a 7-fold increased expression of CD33 on peripheral monocytes [52] which is much higher than what we observed in p.P522R-carriers. Possibly, a limited reduction of phagocytic activity, resulting from a slightly increased CD33 expression, may be advantageous, while an extreme reduction of phagocytic activity, following a strongly increased CD33 expression may have pathogenic effects. To what extent a slightly increased CD33 expression is dependent on the p.P522R genetic variant remains an open question.

### Increased ROS production per particle in myeloid cells

We found that the FcR-*independent* ROS generation was lowered in p.P522R-carriers compared to non-carriers, and that FcR-*dependent* stimulation of monocytes from p.P522R-carriers led to an attenuated phagocytosis. Our results further suggested that the ROS production per phagocytosed particle was increased in p.P522R-carriers, while overall ROS production was similar between carriers and non-carriers. Previous studies indicated that with increasing age, both the phagocytic capacity (# particles ingested/neutrophil) and oxidative burst are reduced [5, 56, 57]. Phagocytic capacity has been reported to be decreased in aged individuals as well as Alzheimer’s disease patients [58]. Our results are in agreement with a recent report by Maguire et al who showed that phagocytosis of opsonized *E. coli* was reduced in p.P522R mouse microglia and macrophages and in human iPSC-derived microglia compared to wild type *PLCG2*. However, the authors argue that upon prolonged stimulation, the deprivation of PLCγ2 substrate PIP2 may explain the decreased phagocytic activity [21]. Additionally, after a functional comparison between homozygous p.P522R knock-in mice and wild type littermates, Takalo et al suggested that the p.P522R variant potentiates the primary function of PLCγ2 as a PIP2-metabolizing enzyme, which is associated with an increased acute inflammatory response and improved overall survival [14]. However, Takalo et al reported an *increased* phagocytic activity of opsonized *E. coli* in murine p.P522R microglia-like cells (BV2 cell-line) and homozygous knock-in murine macrophages, as determined by pHRodo™ Green signal and percentage of phagocytosing cells [14]. The discrepancies between these studies might be explained by differences in experimental setup, such as differences between species, investigated cell types, between p.P522R zygosity, the timeframe used for measuring phagocytic activity, the evaluated readout, and the concentration of substrate.

The more efficient ROS production we observed upon FcR-mediated stimulation in peripheral monocytes may translate directly to the microglia in the in vivo human brain, which also express FcR. Therefore, the p.P522R variant may protect the brain with a mechanism similar to that observed in peripheral immune cells. Although there is discussion on whether phagocytosis is beneficial or detrimental during Alzheimer’s disease [21, 59], a slightly more efficient clearance of tissue damage and debris in the brain may be beneficial at least in the initial stages of Alzheimer's disease. It remains to be investigated to what extent p.P522R benefits carriers early in life, or whether its protective effect is exerted mostly later in life by, for example, delaying immunosenescence, both in the soma and in the central nervous system.

### No differences in antibody complexity upon COVID vaccination

Upon vaccination against SARS-CoV-2 (in vivo evaluation of p.P522R), vaccine-specific antibody levels did not differ between p.P522R carriers and non-carriers in terms of generated serum levels of IgG, IgM or IgA. As we did not have baseline serum samples, we could not exclude a previous SARS-CoV-2 infection, which may influence the serological response. Including these in a future study may give more insights in the actual increase in antibody levels. Moreover, a future in-depth evaluation of the complexity of the Ag-specific B-cell repertoire, especially the VDJ regions of the BCRs and/or antibodies, is warranted to further investigate differences of B-cell response between p.P522R-carriers and non-carriers.

### No impact on peripheral cytotoxic T cells

Other recent in vivo studies reported that *PLCG2* p.P522R mice have an increased level of CD8 T cells in the parenchyma of the healthy brain, which may enhance the cross talk between innate and adaptive immunity [60]. Possibly because the microglia of p.P522R-carriers have an increased HLA expression and thereby an increased capacity to present antigens to T cells. However, our investigation in the peripheral immune system does not offer support for this hypothesis, as we found that P522R carriers have an unchanged fraction of total CD8 T cells or any of the 13 evaluated CD8 T-cell subsets (various activation/memory stages) compared to non-carriers. If CD8 T cells are present in the healthy brain, it may be due to selective recruitment to this specific location, and that does not necessarily reflect the abundance of CD8 T cells in the periphery.

### Limitations of studies in human subjects

Peripheral blood cells provide an easily obtainable source of primary human cells. However, using human subjects means a heterogenous genetic background as compared to inbred animals or cell lines. Previous studies have reported that -amongst others- the genetic constellation, unique to all individuals, has a profound influence on cell counts [61, 62]. In line with these previous studies, we observed an effect of pedigree on the numbers of circulating immune cells. We excluded that the immune-PRS differed between p.P522R-carriers and non-carriers, suggesting that the observed differences between carriers and non-carriers are not influenced by these common genetic elements. Nevertheless, we acknowledge that both p.P522R-carriers and non-carrier siblings may carry additional rare or common genetic elements that affect immune system function. For example, next to the p.P522R variant in *PLCG2* gene, the DRBI*04 variant in the HLA-gene, mainly expressed on antigen-presenting cells, also is associated with protection against neurodegenerative diseases [63].

## Conclusions

In our experiments, we investigated freshly collected peripheral blood cells derived from human p.P522R-carriers and non-carriers. These measurements require minimal sample handling and/or manipulation, providing valuable novel insights into this complex system while staying as close as possible to the actual situation in the donor. Our findings indicate that the p.P522R variant associates with subtle changes of both adaptive and innate circulating human primary cells of the peripheral immune system, which may provide additional insights into the mechanisms of healthy aging.

## Supplementary Information

**Additional file 1: Text S1.** Translated questionnaire to include donor in Cohort II. **Fig. S1.** Impact of genetic background on numbers of circulating immune cells. Each color and symbol indicate members of one family. Fully open symbols represent a centenarian. Semi-open symbol (orange) represents a sibling of a centenarian. Dashed lines indicate the available age-matched reference values produced with flow cytometry panels highly similar to the panels used in this study (earlier or later prototypes of these panels). For B- and T-cell subsets, reference lines indicate a cohort aged 60–79 years. For innate myeloid populations, reference lines indicate a cohort > 55 years old. In plots without dashed lines, no published reference values from highly similar flow cytometry panels were available. **Fig. S2.** Results of the pilot study to evaluate the calcium flux upon stimulation of the B-cell receptor (BCR) with IgG and IgM Fab fragments. (A) Measurement of calcium release (‘flux’) after B-cell stimulation with IgM Fabs in pre-GC B cells (CD27-IgA-IgG-) or unswitched memory B cells (CD27+IgA-IgG-). (B) Measurement of calcium release (‘flux’) after B-cell stimulation with IgG Fabs in CD27-IgG+ memory B cells (CD27-IgD-IgA-) or CD27+IgG+ memory B cells (CD27+IgD-IgA-). Ionomycin was added to calculate maximum calcium release. *N* = 14. Pre-GC; pre-Germinal Center, MBC; memory B cell. **Fig. S3.** Assessment of B-cell activation in all p.P522R-carriers and non-carriers upon BCR stimulation. Measurement of calcium release (‘flux’) after B-cell stimulation with IgM Fabs in pre-GC B cells (CD27-IgG-IgA-) (A) or unswitched memory B cells (CD27+IgG-IgA-) (B) in cohort I. Ionomycin was added to calculate maximum calcium release. Differences between carriers and non-carriers were evaluated by comparing the area under the curve (AUC) of the total Fab stimulation (from stimulation until the moment ionomycin was added, ~ 10 min, flux intensity and duration), the peak of the response after Fab stimulation (the 5 highest points after the Fabs were added to the cells; flux intensity), and after ionomycin was added (to determine the maximum flux). AUC was calculated only for points that were higher than baseline value (unstimulated sample). *N* = 31 (two samples were lost due to technical failure). No significant differences were observed. Pre-GC; pre-Germinal Center. **Fig. S4.** Phagocytosis and ROS production in innate immune cell subsets after stimulation with pHRodo™ Green *E. coli* Bioparticles (FcR/PLCγ2-dependent stimulation). To evaluate the outcome of the phagocytosis assays, three different readouts were used per population: % of cells that were phagocytosing, the average amount of particles phagocytosed per cell, and the ROS production upon phagocytosis. These three readouts were further combined into one value: the normalized ROS. These values are presented for the CD62L+ classical monocyte (cMo) subset (A), intermediate monocytes (iMo) (B), neutrophils (C) CD14- and CD14dim myeloid dendritic cells (mDCs) (D,E), and non-phagocytosing plasmacytoid dendritic cells (pDCs) (F). Lastly, the outcomes for T cells (negative control) are shown (G). Mann-Whitney U test was used to evaluate differences between carriers and non-carriers, but no statistically significant differences were found. *N* = 14. In two donors, monocytes could not be divided into subsets due to absence of a differentiating antibody in the prepared antibody cocktail, therefore, in panel A and B, only 5 non-carriers and 7 carriers are shown. Dashed lines indicate the background level of ROS (ROS production in negative control population; T cells). All outcomes were corrected for background or baseline activation by subtracting the value of the control (incubated on ice) from the activated (incubated at 37 °C) sample. Negative values (caused by higher background in control samples than activated samples in cell populations that did not perform phagocytosis) were set to 0. **Fig. S5.** ROS generation in innate immune cell subsets in p.P522R-carriers and non-carriers upon stimulation with PMA (FcR/PLCγ2-independent stimulation). *N* = 14. In two donors, monocytes could not be divided into subsets due to absence of an antibody in the prepared antibody cocktail, therefore, in panel **A-D**, only 5 non-carriers and 7 carriers are shown. Dashed lines indicate the background level of ROS (ROS production in negative control population; T cells). All outcomes were corrected for background or baseline activation by subtracting the value of the control (incubated on ice) from the activated (incubated at 37 °C) sample. **Table S1.** Description of all included families and additional donors. **Table S2.** Overview of the flow cytometry panels that were used in this study. **Table S3.** Phenotypic descriptions used to define B-cell subsets stained with EuroFlow PERISCOPE B-cell and plasma cell panel (BIGH) panel by manual analysis. **Table S4.** Phenotypic descriptions used to define T-cell subsets stained with EuroFlow PERISCOPE CD4 T-cell (TCD4) panel by manual analysis. **Table S5.** Phenotypic descriptions used to define T-cell and NK-cell subsets stained with the EuroFlow PERISCOPE CD8 cytotoxic T-cell (CYTOX) panel by manual analysis. **Table S6.** Phenotypic descriptions used to define innate immune cell (sub) sets stained with the EuroFlow PERISCOPE DC-Monocyte panel by manual analysis. **Table S7.** Polygenic Risk Score and SNP annotation for immune-related SNPs. **Table S8.** Control and assay tubes measured in the phagocytosis experiment. **Table S9.** Control and assay tubes measured when detection production of reactive oxygen species (ROS).

## Data Availability

The datasets used and/or analysed during the current study are available from the corresponding authors on reasonable request. Questions and requests about the clinical data should be submitted to H. Holstege. Questions and requests for the experimental data discussed in this manuscript should be submitted to J.J.M. van Dongen.

## Abbreviations

PLCγ2: Phospholipase C gamma 2
BCR, TCR: B and T-cell receptor
GC: Germinal center
MBC: Memory B cell
ROS: Reactive oxygen species
SNP: Single-nucleotide polymorphism
*PLCG2*: Phospholipase C gamma 2 gene
PIP2: Phosphatidylinositol 4,5-bisphosphate
IP3: Inositol 1,4,5-triphosphate
DAG: Diacylglycerol
FcR: Fc receptors
TREM: Triggering Receptors Expressed on Myeloid cells
PB: Peripheral blood
GSA: Genome Screening Array
QC: Quality control
CS&T beads: Cytometer Setup and Tracking beads
DC-monocyte: Dendritic cell-monocyte
CD4T: CD4-T cell panel
CYTOX: CD8 cytotoxic T-cell panel
BIGH: B-cell and plasma cell panel
PRS: Polygenic risk score
GWAS: Genome-wide assocation study
MIA: Multiplex immunoassay
AD: Alzheimer's disease
FDR: False Discovery Rate
PBMCs: Peripheral blood mononuclear cells
AUC: Area under the curve
Cj: Coding joints
Sj: Signal joints
Alb: Albumin
DHR123: Dihydrorhodamine
cMos, iMos and ncMos: Classical, intermediate, and non-classical monocytes
pPLCγ2: Phosphorylated PLCγ2
iMFI: Integrated mean fluorescent intensity
KREC: Kappa-recombination excision circle
RBD: Receptor Binding Domain
S protein: Spike protein
N protein: Nucleocapsid protein
mDCs, pDCs: Myeloid and plasmacytoid dendritic cells
PMA: Phorbol 12-Myristate 13-acetate
NSAIDs: Non-steroid anti-inflammatory drugs
