# Supplementary material for: Carriers of the p.P522R variant in PLCγ2 have a slightly more responsive immune system
Source: Mol Neurodegener. 2023 Apr 20;18:25. doi: 10.1186/s13024-023-00604-9 (PMC10116473; doi:10.1186/s13024-023-00604-9)
Supplement: Supplementary file 1 — Additional file 1: Text S1. Translated questionnaire to include donor in Cohort II. Fig. S1. Impact of genetic background on numbers of circulating immune cells. Each color and symbol indicate members of one family. Fully open symbols represent a centenarian. Semi-open symbol (orange) represents a sibling of a centenarian. Dashed lines indicate the available age-matched reference values produced with flow cytometry panels highly similar to the panels used in this study (earlier or later prototypes of these panels). For B- and T-cell subsets, reference lines indicate a cohort aged 60–79 years. For innate myeloid populations, reference lines indicate a cohort > 55 years old. In plots without dashed lines, no published reference values from highly similar flow cytometry panels were available. Fig. S2. Results of the pilot study to evaluate the calcium flux upon stimulation of the B-cell receptor (BCR) with IgG and IgM Fab fragments. (A) Measurement of calcium release (‘flux’) after B-cell stimulation with IgM Fabs in pre-GC B cells (CD27-IgA-IgG-) or unswitched memory B cells (CD27+IgA-IgG-). (B) Measurement of calcium release (‘flux’) after B-cell stimulation with IgG Fabs in CD27-IgG+ memory B cells (CD27-IgD-IgA-) or CD27+IgG+ memory B cells (CD27+IgD-IgA-). Ionomycin was added to calculate maximum calcium release. N = 14. Pre-GC; pre-Germinal Center, MBC; memory B cell. Fig. S3. Assessment of B-cell activation in all p.P522R-carriers and non-carriers upon BCR stimulation. Measurement of calcium release (‘flux’) after B-cell stimulation with IgM Fabs in pre-GC B cells (CD27-IgG-IgA-) (A) or unswitched memory B cells (CD27+IgG-IgA-) (B) in cohort I. Ionomycin was added to calculate maximum calcium release. Differences between carriers and non-carriers were evaluated by comparing the area under the curve (AUC) of the total Fab stimulation (from stimulation until the moment ionomycin was added, ~ 10 min, flux intensity and duration), the peak of the response after Fab [file 13024_2023_604_MOESM1_ESM.zip › Diks et al. PCLG2 P522R and immune system_supplementary.docx]

Additional Files/Supplementary Materials for

**Carriers of the p.P522R variant in PLCγ2 have a slightly more responsive immune system**

Annieck M. Diks^1,2^, Cristina Teodosio^1,3^, Bas de Mooij^1^, Rick J. Groenland^1^, Brigitta A.E. Naber^1^, Inge F. de Laat^1^, Sandra A. Vloemans^1^, Susan Rohde^2^, Marien I. de Jonge^4^, Linda Lorenz^2^, Debbie Horsten^2^, Jacques J.M. van Dongen^1,3,*^, Magdalena A. Berkowska^1†^, Henne Holstege^2†*^

† These authors contributed equally to this work.

* Correspondence clinical work: H. Holstege ([H.Holstege@amsterdamumc.nl](mailto:H.Holstege@amsterdamumc.nl))

* Correspondence laboratory work: J.J.M. van Dongen ([J.J.M.van_Dongen@lumc.nl](mailto:J.J.M.van_Dongen@lumc.nl))

**This file includes:**

Supplementary Text

Figs. S1 to S5

Tables S1 to S9 (Table S7 is a separate excel file)

Supplementary Text

Translated questionnaire to include donor in Cohort II
Of note, this file was translated by the authors for the sole purpose of transparency.

**General health** **evaluation***
Are you currently, or were you in the past diagnosed with:

1. Hypertension?
   1. If so, please specify age and medication
2. Diabetes type 2?
   1. If so, please specify age and medication
3. Cardiovascular issues?
   1. If so, please specify the type of issue (cardiovascular infarct, angina pectoris, arrhythmia, cardiac valve issue, cardiovascular failure, other: ...)
   2. Please indicate if you were treated by a specialist and which treatment was received
4. A brain infarct?
   1. If so, please specify (Cerebrovascular attack, Transcient Ischemic Attack or ‘other’)
   2. If so, please specify age
   3. Please indicate if you were treated by a specialist
5. An autoimmune disease
   1. If so, please specify which autoimmune disease
   2. If so, please specify age
   3. (medication is recorded later in the questionnaire)
6. Osteoporosis
   1. If so, please specify age
7. A tumor or cancer
   1. If so, please specify the type of tumor/cancer
   2. If so, please specify age at time of tumor/cancer
   3. If so, please specify which treatment was received
8. Do you have any allergies?
   1. If so, please specify

**Gastro-intestinal Health***

1. Do you currently experience diarrhea?
   1. If so, please specify date of onset
2. Do you currently experience Irritable Bowel Disease (IBD)?
   1. If so, please specify date of onset
3. Are you diagnosed with Colitus Ulcerosa?
   1. If so, please specify date of onset
   2. If so, are you currently experiencing an episode?
4. Are you currently diagnosed with any other gastro-intestinal disease?
   1. If so, is diarrhea one of the symptoms?
5. Are you currently diagnosed with a stomach ulcer?
   1. If so, please specify date of onset

**Additional questions to determine if a person was eligible for blood donation within a short timeframe****

1. Do you currently experience an (active) infection?
   1. If so, please specify which type of infection
2. Did you experience an (active) infection during the past three months?
   1. If so, please specify which type of infection
   2. If so, please specify the date and duration of the infection
3. Have you been diagnosed with SARS-CoV2 infection?
   1. If so, please specify the date
   2. If so, please indicate the last day of symptoms
4. Has someone in your household been diagnosed with SARS-CoV2 infection?
   1. If so, please specify the date
   2. If so, please indicate the last day of symptoms
5. Has one of your direct caregivers been diagnosed with SARS-CoV2 infection?
   1. If so, please specify the date
   2. If so, please indicate the last day of symptoms
6. When living in an elderly home; have there been SARS-CoV2 infections in the elderly home?
   1. If so, please specify the date
7. Did you receive a vaccination in the past 6 months?
   1. If so, please specify type of vaccine and date of vaccination
8. Did you receive a vaccination in the past 3 weeks?
   1. If so, please specify type of vaccine and date of vaccination
9. Do you have any vaccination planned soon?
   1. If so, please specify date
10. Are you currently a blood donor, or were you until very recently a blood donor?
    1. If so, please specify which timeframe and date of last blood donation
11. Did you undergo major surgery in the past month?
    1. If so, please specify
12. Do you experience another health conditions that was not discussed in this questionnaire?

**Use of medication and vitamins***

1. Do you currently use antibiotics?
   1. If so, please specify which antibiotics and what timeframe
2. Do you currently use corticosteroids?
   1. If so, please specify which corticosteroids and what timeframe
3. Do you currently use other medication?
   1. If so, please specify which and what timeframe
4. Do you currently use vitamin supplements?
   1. If so, please specify which vitamins and what timeframe

* Most sections in this questionnaire were part of the general questionnaire asked upon inclusion in cohort I. However, for inclusion into Cohort II, these were asked once again in combination with an additional set of questions, as indicated in this text with a double asterisk (**).


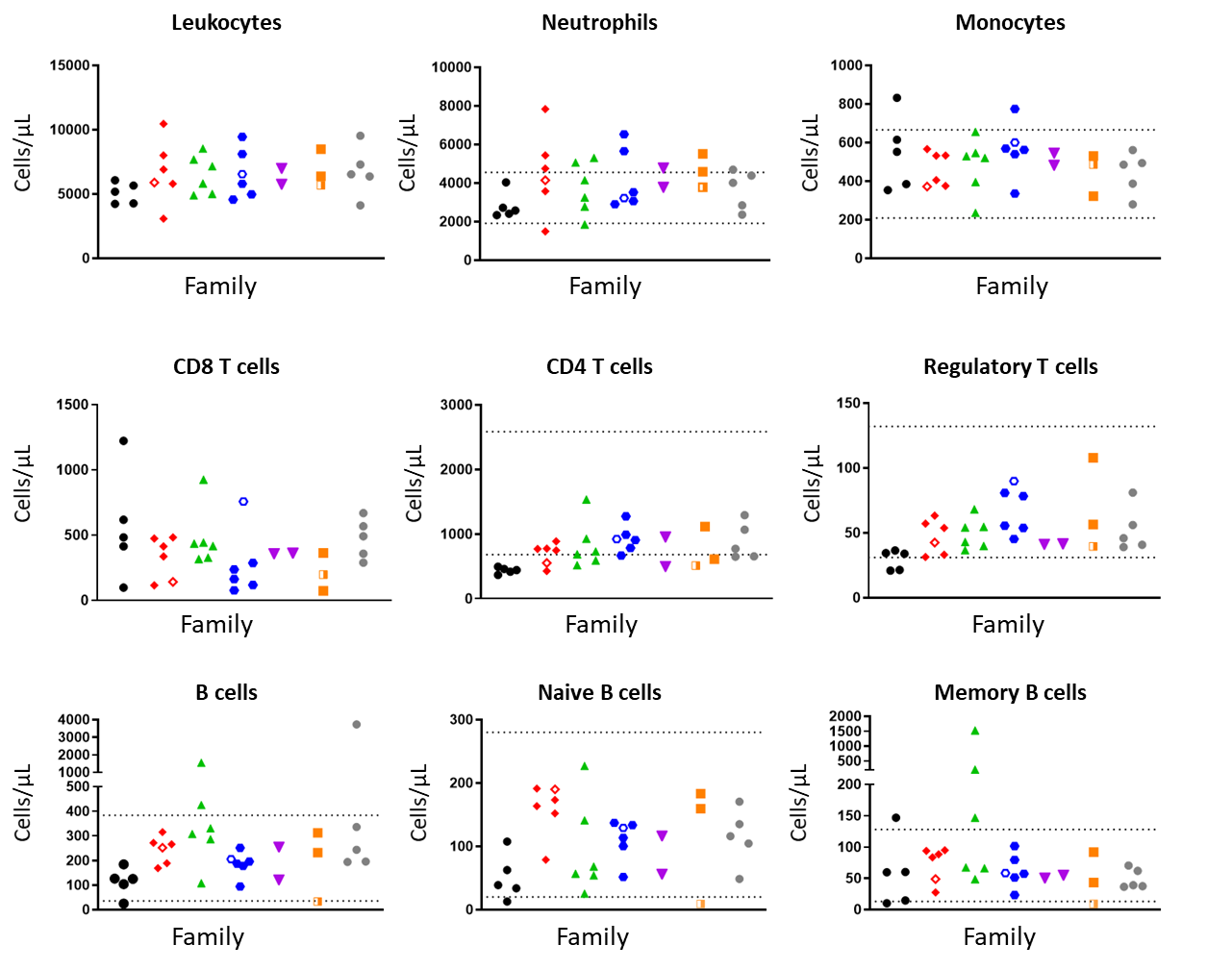


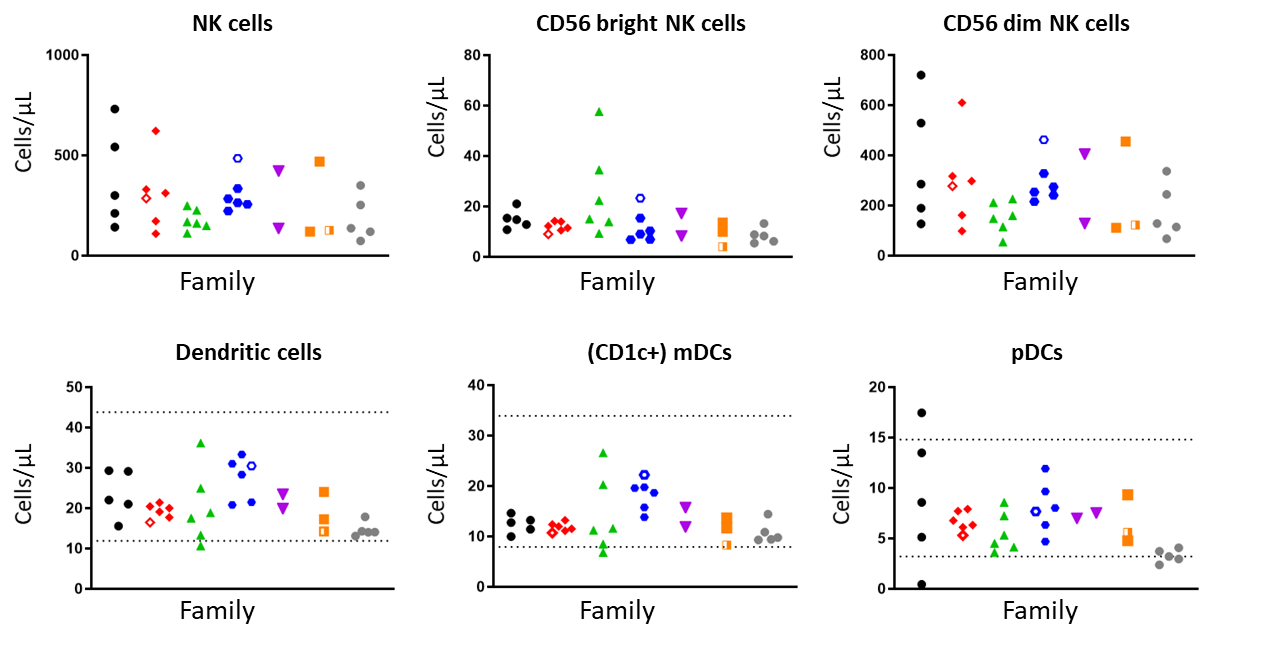


Fig. S1.

Impact of genetic background on numbers of circulating immune cells. Each color and symbol indicate members of one family. Fully open symbols represent a centenarian. Semi-open symbol (orange) represents a sibling of a centenarian. Dashed lines indicate the available age-matched reference values produced with flow cytometry panels highly similar to the panels used in this study (earlier or later prototypes of these panels). For B- and T-cell subsets, reference lines indicate a cohort aged 60-79 years. For innate myeloid populations, reference lines indicate a cohort >55 years old. In plots without dashed lines, no published reference values from highly similar flow cytometry panels were available.


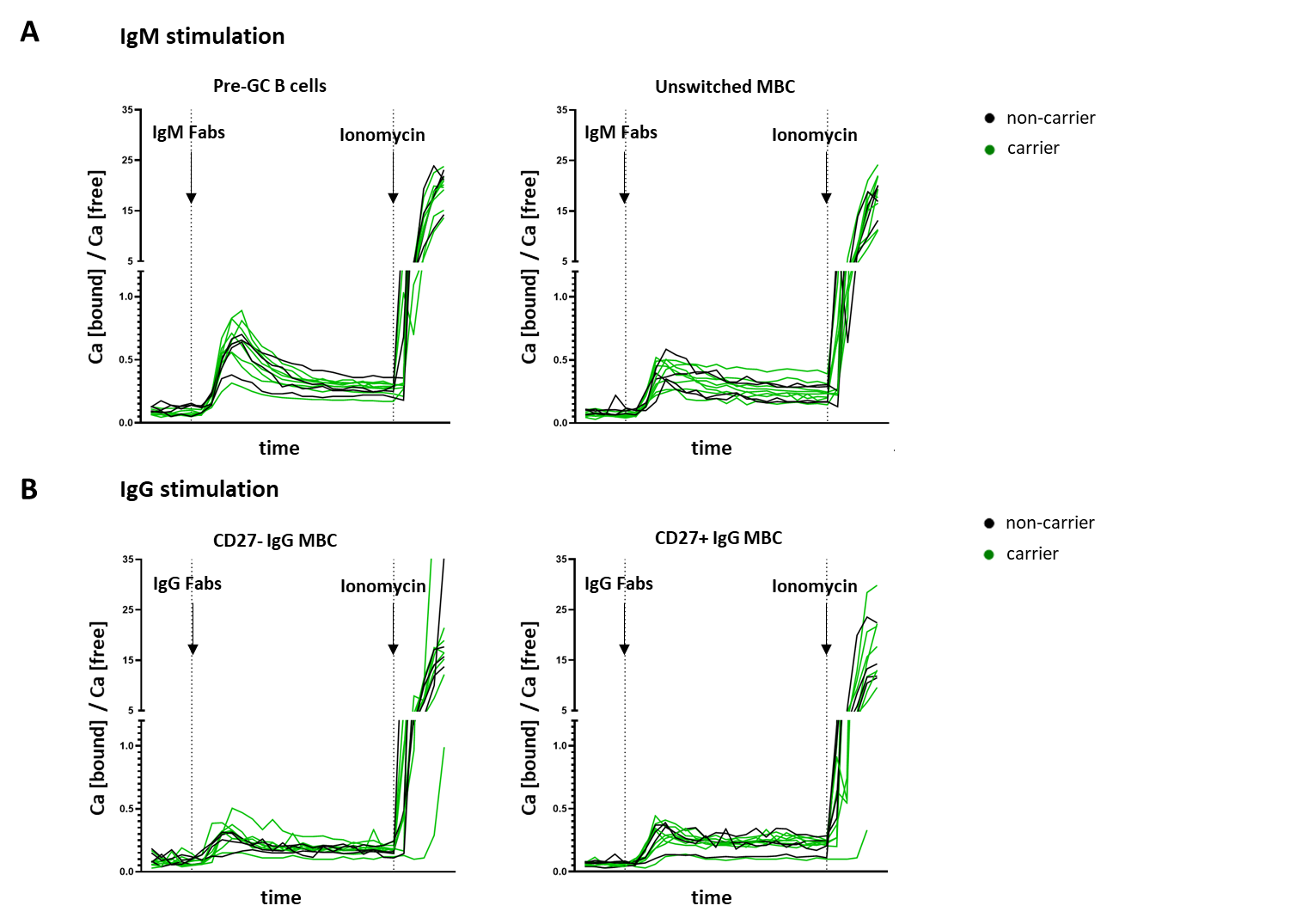


Fig. S2.

Results of the pilot study to evaluate the calcium flux upon stimulation of the B-cell receptor (BCR) with IgG and IgM Fab fragments. (A) Measurement of calcium release (‘flux’) after B-cell stimulation with IgM Fabs in pre-GC B cells (CD27-IgA-IgG-) or unswitched memory B cells (CD27+IgA-IgG-). (B) Measurement of calcium release (‘flux’) after B-cell stimulation with IgG Fabs in CD27- IgG+ memory B cells (CD27-IgD-IgA-) or CD27+IgG+ memory B cells (CD27+IgD-IgA-). Ionomycin was added to calculate maximum calcium release. N=14. Pre-GC; pre-Germinal Center, MBC; memory B cell.


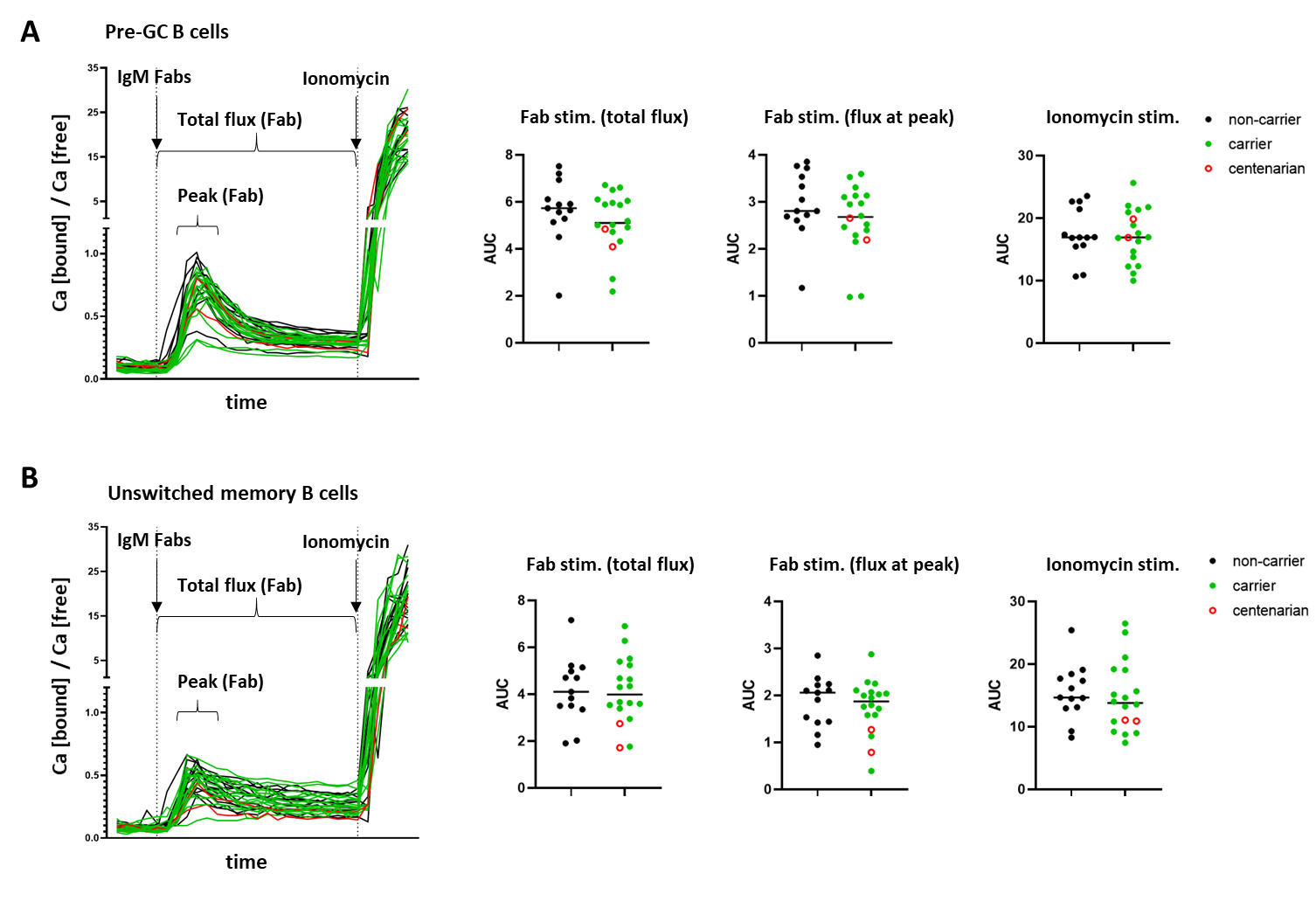


Fig. S3. Assessment of B-cell activation in all p.P522R-carriers and non-carriers upon BCR stimulation. Measurement of calcium release (‘flux’) after B-cell stimulation with IgM Fabs in pre-GC B cells (CD27-IgG-IgA-) (A) or unswitched memory B cells (CD27+IgG-IgA-) (B) in cohort I. Ionomycin was added to calculate maximum calcium release. Differences between carriers and non-carriers were evaluated by comparing the area under the curve (AUC) of the total Fab stimulation (from stimulation until the moment ionomycin was added, ~ 10 min, flux intensity and duration), the peak of the response after Fab stimulation (the 5 highest points after the Fabs were added to the cells; flux intensity), and after ionomycin was added (to determine the maximum flux). AUC was calculated only for points that were higher than baseline value (unstimulated sample). N=31 (two samples were lost due to technical failure). No significant differences were observed. Pre-GC; pre-Germinal Center.


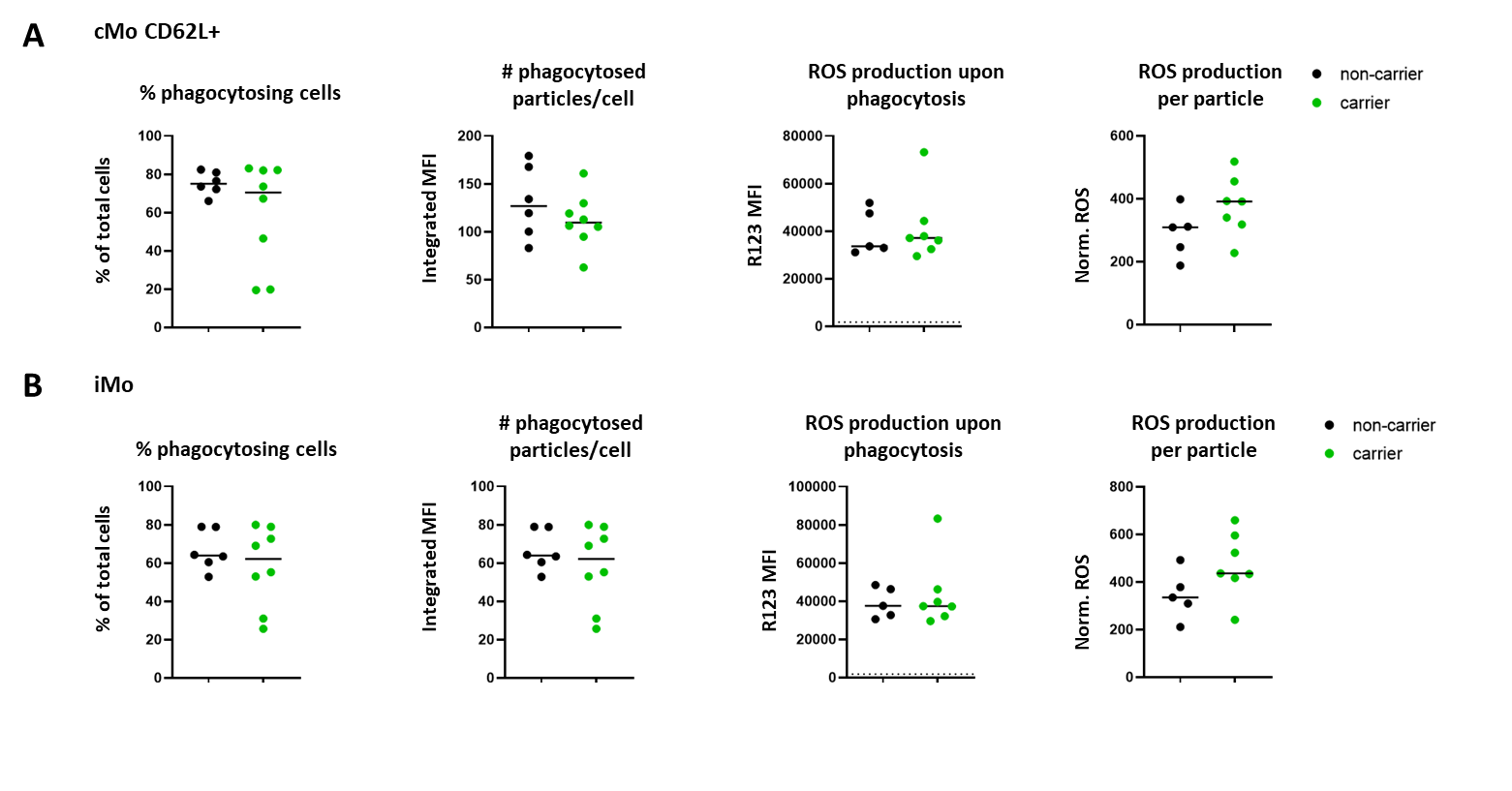


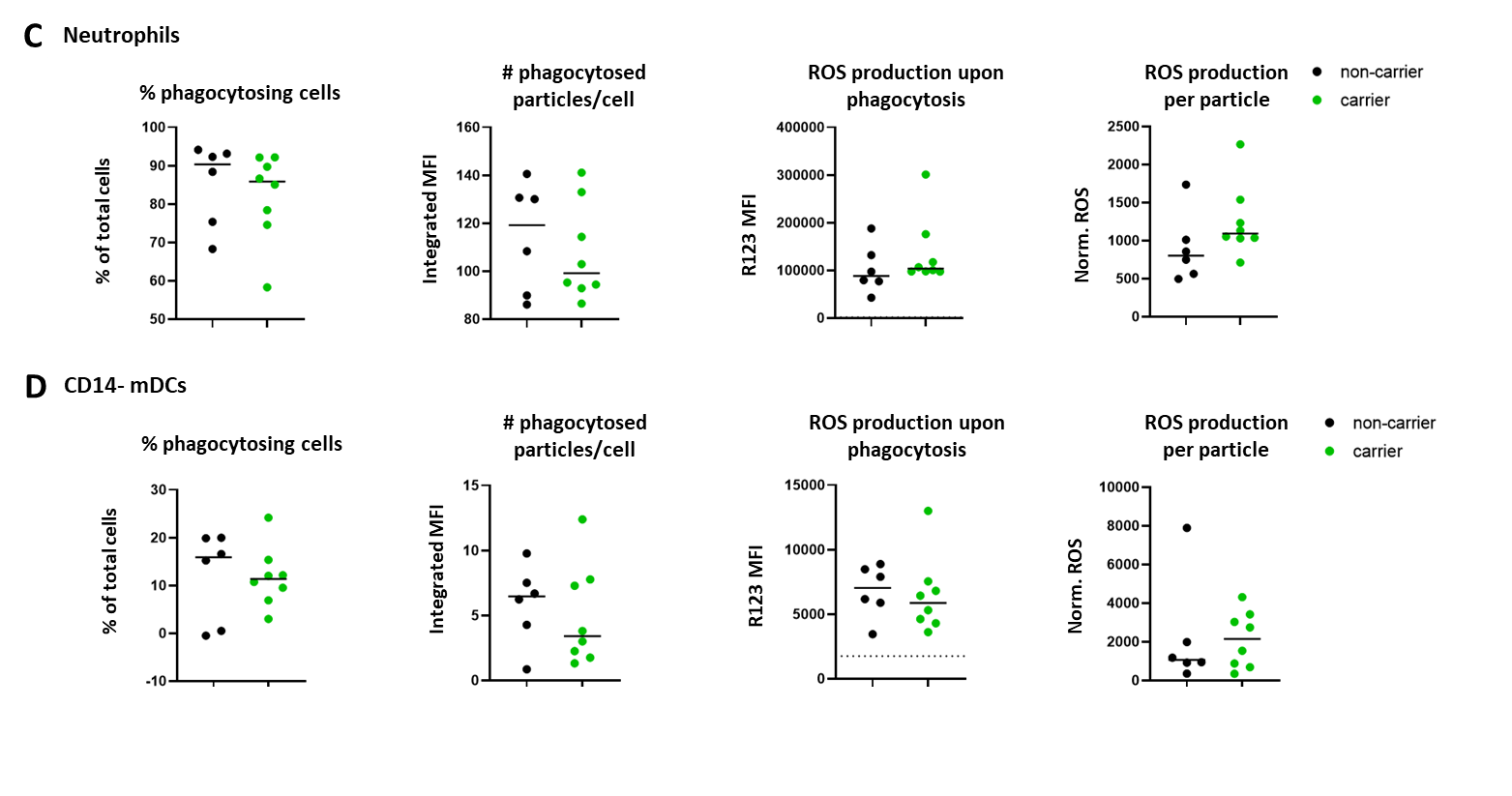


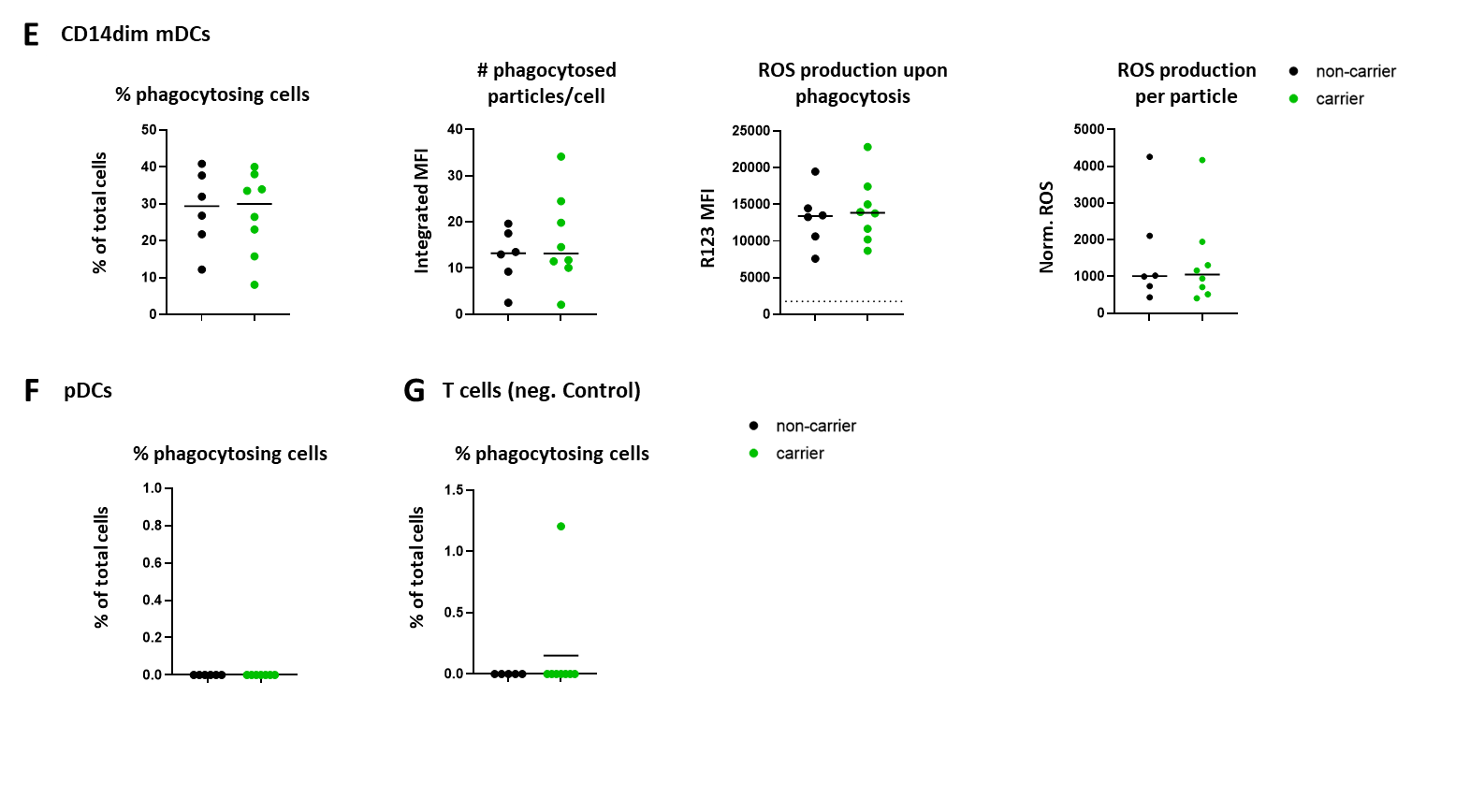


Fig. S4. (on previous page)

Phagocytosis and ROS production in innate immune cell subsets after stimulation with pHRodo™ Green *E. coli* Bioparticles (FcR/PLCγ2-dependent stimulation). To evaluate the outcome of the phagocytosis assays, three different readouts were used per population: % of cells that were phagocytosing, the average amount of particles phagocytosed per cell, and the ROS production upon phagocytosis. These three readouts were further combined into one value: the normalized ROS. These values are presented for the CD62L+ classical monocyte (cMo) subset (A), intermediate monocytes (iMo) (B), neutrophils (C) CD14- and CD14dim myeloid dendritic cells (mDCs) (D,E), and non-phagocytosing plasmacytoid dendritic cells (pDCs) (F). Lastly, the outcomes for T cells (negative control) are shown (G). Mann-Whitney test was used to evaluate differences between carriers and non-carriers, but no statistically significant differences were found. N=14. In two donors, monocytes could not be divided into subsets due to absence of a differentiating antibody in the prepared antibody cocktail, therefore, in panel A and B, only 5 non-carriers and 7 p.P522R-carriers are shown. Dashed lines indicate the background level of ROS (ROS production in negative control population; T cells). All outcomes were corrected for background or baseline activation by subtracting the value of the control (incubated on ice) from the activated (incubated at 37°C) sample. Negative values (caused by higher background in control samples than activated samples in cell populations that did not perform phagocytosis) were set to 0.


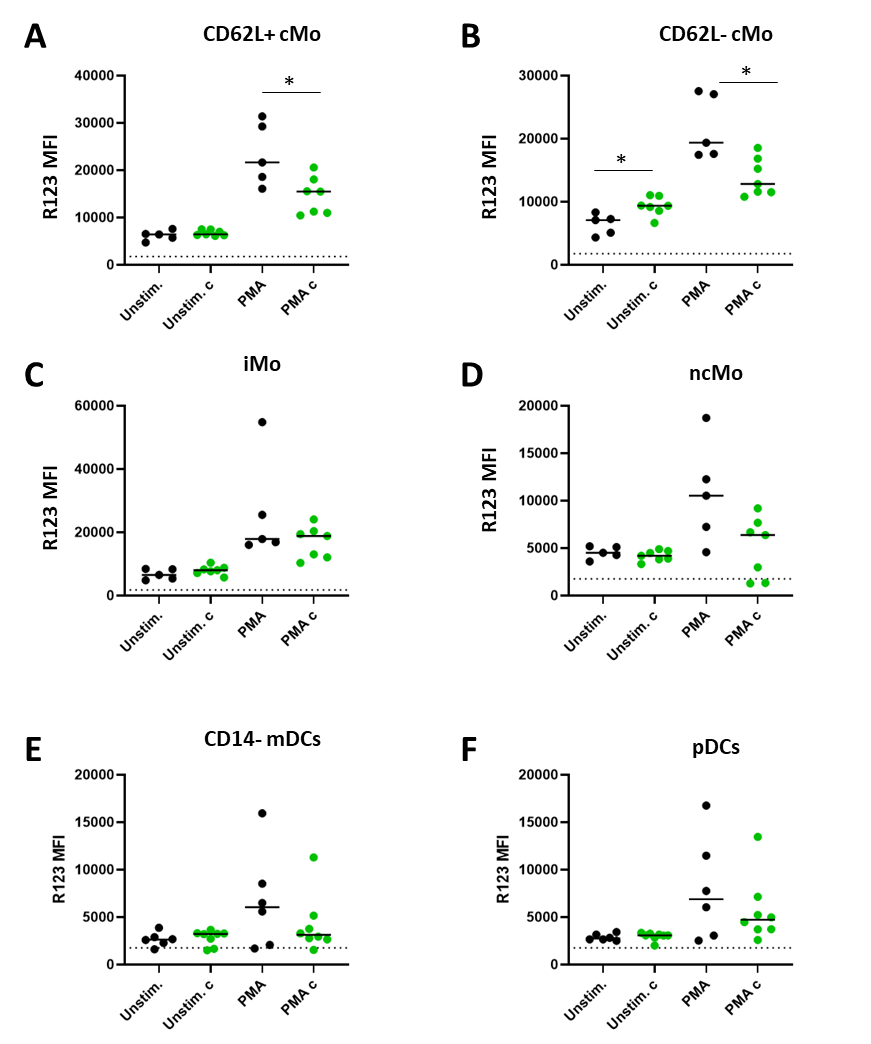


Fig. S5.

ROS generation in innate immune cell subsets in p.P522R-carriers and non-carriers upon stimulation with PMA (FcR/PLCγ2-independent stimulation). N=14. In two donors, monocytes could not be divided into subsets due to absence of an antibody in the prepared antibody cocktail, therefore, in panel A-D, only 5 non-carriers and 7 carriers are shown. Dashed lines indicate the background level of ROS (ROS production in negative control population; T cells). All outcomes were corrected for background or baseline activation by subtracting the value of the control (incubated on ice) from the activated (incubated at 37°C) sample.

Table S1.

Description of all included families and additional donors

| **Family** | **M/F** | **Carrier/**  **non carrier** | **Age range (min-max)** |
| --- | --- | --- | --- |
| **Family 1** | 4/1 | 3/2 | 63-75 years |
| Additional: sibling of the centenarian | F | Carrier | 94 years |
| **Family 2** | 3/2 | 1/4 | 62-83 years |
| **Family 3** | 2/4 | 5/1 | 67-79 years |
| **Family 4** | 1/1 | 1/1 | 72-74 years |
| Additional: the centenarian | F | Carrier | 103 years |
| **Family 5** | 2/3 | 2/3 | 66-75 years |
| Additional: the centenarian | F | Carrier | 103 years |
| **Family 6** | 1/1 | 1/1 | 65-81 years |
| **Family 7** | 1/4 | 2/3 | 59-78 years |
| **Additional donor 1** | F | Carrier | 71 years |
| **Additional donor 2** ^†^ | M | Carrier | 69 years |
| **Additional donor 3** | F | Non-carrier | 71 years |

M= male, F= female

^†^ Exceptionally, one donor (carrier) was included from a family with history of healthy aging, but the parent was not yet a centenarian (93 years old at time of inclusion)

Table S2. Overview of the flow cytometry panels that were used in this study (table continues on the next pages).

| **Flow cytometry panel** | **Antibody** | | **clone** | **provider** | **titer (ul)** | **Surface stain** | | **Intracellular stain** | |
| --- | --- | --- | --- | --- | --- | --- | --- | --- | --- |
| **BIGH panel (B-cell and plasma cell panel)** | CD27 BV421 | | M-T27156 | BD | 2 | x | |  | |
| **10*10^6 cells stained** | IgM BV510 | | MHM-88 | BioLegend | 2 | x | | x | |
|  | CD62L BV605 | | DREG-56 | BioLegend | 5 | x | |  | |
| **EuroFlow protocols used:** | CD24 BV650 | | ML5 | BD | 5 | x | |  | |
| **1. Bulk lysis** | CD21 BV711 | | B-Ly4 | BD | 5 | x | |  | |
| **2. Intracellular stain (Fix & Perm)** | CD19 BV786 | | SJ25C1 | BD | 4 | x | |  | |
| **Official reference for the BIGH panel:**  Blanco E, Pérez-Andrés M, Arriba-Méndez S, et al. Age-associated distribution of normal B-cell and plasma cell subsets in peripheral blood. *JACI* 2018;141(6):2208-2219. e2216.  Diks AM, Versteegen P, Teodosio C, et al. Age and Primary Vaccination Background Influence the Plasma Cell Response to Pertussis Booster Vaccination. *Vaccines.* 2022;10(2):136.  Patent filed by Van Dongen et al. Means and Methods for Multiparameter Cytometry-Based Leukocyte Subsetting. P119646NL00 (2019). PCT/NL2020/050688, priority date 5 November 2019. | Ig Subclasses kit (CAT: CYT-IGS-1) | | n/a | Cytognos | 25 | x | | x | |
|  | CD20 PE CF594 | | 2H7 | BD | 2.5 (1:10) | x | |  | |
|  | CD138 PE-Cy7 | | MI15 | BioLegend | 5 | x | |  | |
|  | CD5 PE-Cy7 | | LIF7F12 | BD | 6 | x | |  | |
|  | IgD APC | | IA6-2 | BD | 4 | x | | x | |
|  | CD38 APC H7 | | HB7 | BD | 3 | x | |  | |
|  | | | | | | | | | |
| **DC-Monocyte panel (dendritic cell- monocyte panel)** | CD141 BV421 | | 1A4 | BD | 2.5 | x | | no intracellular staining | |
| **10*10^6 cells stained** | CD45 OC515 | | GA90 | Cytognos | 10 | x | |  |  |
|  | CD62L BV605 | | DREG-56 | BioLegend | 5 | x | |  |  |
| **EuroFlow protocols used:** | HLA-DR BV711 | | G46-6 | BD | 2.5 | x | |  |  |
| **1. Bulk lysis** | CD16 BV786 | | 3G8 | BD | 5 | x | |  |  |
| **Official reference for the IMC panel:**  Van der Pan et al, Development of a standardized and validated flow cytometry approach for monitoring of innate myeloid immune cells in human blood, Frontiers in Immunology, 2022, 5141  Patent filed by Van Dongen et al. Means and Methods for Multiparameter Cytometry-Based Leukocyte Subsetting. P119646NL00 (2019). PCT/NL2020/050688, priority date 5 November 2019. | CD1c BB515 | | F10/21A3 | BD | 5 | x | |  |  |
|  | CD36 PerCP Cy5.5 | | CLB-IVC7 | Immunostep | 10 | x | |  |  |
|  | SLAN PE | | DD.1 | Miltenyi | 10 | x | |  |  |
|  | FcERI PE | | AER-37 | Thermo Fischer | 5 | x | |  |  |
|  | CD33 PE-Cy7 | | P67.6 | BD | 5 | x | |  |  |
|  | CD300 (IREM2) APC | | UP-H2 | Immunostep | 10 | x | |  |  |
|  | CD303 APC | | AC144 | Miltenyi | 10 | x | |  |  |
|  | CD14 APC H7 | | MφP9 | BD | 5 | x | |  |  |
|  | Brilliant Stain buffer | | n/a | BD | 50 | x | |  |  |
|  | | | | | | | | | |
| **CD4T panel (CD4 T cells)** | CD27 BV421 | | M-T27156 | BD | 2 | | x | | no intracellular staining |
| **100ul peripheral blood stained** | CD45RA BV510 | | HI100 | BD | 2.5 | | x | |  |
|  | CD62L BV650 | | DREG-56 | BioLegend | 2.5 | | x | |  |
| **EuroFlow protocols used:** | CD127 BV711 | | HIL7RM21 | BD | 5 | | x | |  |
| **1. Stain Lyse Wash** | CD3 BV786 | | SK7 | BD | 1 | | x | |  |
| **Official reference for the CD4T panel:**  Botafogo V, Pérez-Andres M, Jara-Acevedo M, et al. Age distribution of multiple functionally relevant subsets of CD4+ T cells in human blood using a standardized and validated 14-color EuroFlow immune monitoring tube. *Frontiers in immunology.* 2020;11:166.  Patent filed by Van Dongen et al. Means and Methods for Multiparameter Cytometry-Based Leukocyte Subsetting. P119646NL00 (2019). PCT/NL2020/050688, priority date 5 November 2019. | CD25 VioBright FITC | | 4E3 | Milentyi | 10 | | x | |  |
|  | CCR10 PerCP Cy5.5 | | 1B5 | BD | 2.5 | | x | |  |
|  | CXCR3 PE | | 1C6/CXCR3 | BD | 10 | | x | |  |
|  | CCR6 PE-CF594 | | 11A9 | BD | 5 | | x | |  |
|  | CCR4 PE-Cy7 | | L291H4 | BioLegend | 1 | | x | |  |
|  | CXCR5 APC | | 51505 | R&D | 10 | | x | |  |
|  | CD4 APC H7 | | SK3 | BD | 5 | | x | |  |
|  | |  | | | | | | | |
| **CYTOX panel (CD8 cytotoxic T-cell panel)** | CD27 BV421 | | M-T27156 | BD | 2 | | x | |  |
| **100ul peripheral blood stained** | CD45RA BV510 | | HI100 | BD | 2.5 | | x | |  |
|  | CD62L BV650 | | DREG-56 | BioLegend | 2.5 | | x | |  |
| **EuroFlow protocols used:** | CD16 BV711 | | 3G8 | BD | 2.5 | | x | |  |
| **1. Stain Lyse** | CD3 BV786 | | SK7 | BD | 1 | | x | |  |
| **2. Intracellular stain (Fix & Perm)** | CD57 FITC | | HNK1 | BD | 10 | | x | |  |
| **Official reference for the CYTOX panel:**  Patent filed by Van Dongen et al. Means and Methods for Multiparameter Cytometry-Based Leukocyte Subsetting. P119646NL00 (2019). PCT/NL2020/050688, priority date 5 November 2019. | CD28 PerCP Cy5.5 | | CD28.2 | BioLegend | 5 | | x | |  |
|  | Granzyme B PE | | GB11 | Sanquin | 15 | | - | | x |
|  | CD8 PE-CF594 | | RPAT8 | BD | 1 | | x | |  |
|  | TCRgd PE-Cy7 | | 11F2 | BD | 1 | | x | |  |
|  | CD56 APC H7 | | HCD56 | BioLegend | 5 | | x | |  |

| **Flow cytometry panel** | **Antibody** | **clone** | **provider** | **titer (ul)** | **Surface stain** | **Intracellular stain** |
| --- | --- | --- | --- | --- | --- | --- |
| **PLCγ2 panel** | CD27 BV421 | M-T27156 | BD | 2 | x |  |
| **2.5 * 10^5 cells stained** | IgM BV510 | MHM-88 | Biolegend | 2 | x |  |
|  | CD45 BV650 | ML5 | BD | 20 | x |  |
| **EuroFlow protocols used:** | CD19 BV786 | SJ25C1 | BD | 4 | x |  |
| **1. Bulk lysis** | IgA FITC | IS11-8E10 | Miltenyi | 1 | x |  |
| **2. Intracellular stain (Fix & Perm)** | IgD PerCP Cy5.5 | IA6-2 | Biolegend | 5 | x |  |
|  | IgG PE | G18-145 | BD | 20 | x |  |
|  | CD20 PE CF594 | 2H7 | BD | 2.5 (1:10) | x |  |
|  | CD3 PE Cy7 | SK7 | BD | 5 | x |  |
|  | CD138 PE Cy7 | MI15 | Biolegend | 5 | x |  |
|  | PLCg2 APC | REA488 | Miltenyi | 15 | - | x |
|  | CD16 AF700 | 3G8 | BD | 5 | x |  |
|  | CD38 APC H7 | HB7 | BD | 3 | x |  |
|  | | | | | | |
| **Perfect Count** | CD45 OC515 | GA90 | Cytognos | 5 | x | no intracellular staining |
| **50μl peripheral blood stained** | CD19 BV786 | SJ25C1 | BD | 4 | x |  |
| **EuroFlow protocol used: PerfectCount** | CD3 APC | SK7 | BD | 2.5 | x |  |

| **Flow cytometry panel** | **Antibody** | **clone** | **provider** | **titer (ul)** | **Surface stain** | **Intracellular stain** |
| --- | --- | --- | --- | --- | --- | --- |
| **PLCγ2 Phosphorylation** | CD20 PE Cy7 | 2H7 | BD | 5 | x |  |
| **2.5 * 10^5 cells stained** | CD3 FITC | SK7 | BD | 20 | x |  |
|  | CD16 BV510 | 3G8 | BD | 10 | x |  |
| **Custom protocol; see Methods section** | CD27 BV421 | M-T271 | BD | 2 | x |  |
|  | Zombie NIR™ Fixable Viability Kit | n/a | Biolegend | 1 (1:10) | x |  |
|  | pPLCg2 APC | REA341 | Miltenyi | 10 | - | x |
|  | IgG PE | G18-145 | BD | 10 | - | x |
|  | IgA PE | IS11-8E10 | Miltenyi | 4 | - | x |
|  | | | | | | |
| **Caflux** | Zombie NIR™ Fixable Viability Kit | n/a | Biolegend | 1 (1:10) | x | No intracellular staining |
| **2.5 * 10^5 thawed peripheral blood mononuclear cells (PBMCs) stained** | IgA FITC | IS11-8E10 | Miltenyi | 1 | x |  |
|  | IgG PE | G18-145 | BD | 10 | x |  |
| **Custom protocol; see Methods section** | IgD PerCP Cy5.5 | IA6-2 | Biolegend | 5 | x |  |
|  | CD20 PE-Cy7 | 2H7 | BD | 5 | x |  |
|  | CD27 BV421 | M-T271 | BD | 2 | x |  |

| **Flow cytometry panel** | **Antibody** | **clone** | **provider** | **titer (ul)** | **Surface stain** | **Intracellular stain** |
| --- | --- | --- | --- | --- | --- | --- |
| **PhagoBURST + Phagocytosis assay** | CD141 BV421 | 1A4 | BD Biosciences | 2.5 | x | No intracellular staining |
| **200ul peripheral blood stained** | CD15 BV510* | W6D3 | BD biosciences | 15 | x |  |
|  | CD19 BV605 | HIB19 | Biolegend | 5 | x |  |
| **Custom protocol; see Methods section** | CD62L BV650 | DREG-56 | Biolegend | 2.5 | x |  |
|  | HLA DR BV786 | G46-6 | Bd Biosciences | 2.5 | x |  |
|  | CD16 BV786 | 3G8 | BD biosciences | 5 | x |  |
|  | SLAN PE | DD.1 | Miltenyi | 5 | x |  |
|  | CD36 PerCP Cy5.5 | CLB-IVC7 | Immunostep | 10 | x |  |
|  | CD33 PE Cy7 | P67.6 | BD biosciences | 5 | x |  |
|  | CD14 PE CF594 | MφP9 | BD biosciences | 5 | x |  |
|  | IREM-2 APC | UP-H2 | Immunostep | 10 | x |  |
|  | CD303 APC | AC144 | Miltenyi | 5 | x |  |
|  | CD45 AF700 | HI30 | BD biosciences | 5 | x |  |
|  | CD3 APC H7 | SK7 | BD Biosciences | 5 | x |  |
|  | Brilliant Stain buffer | n/a | BD Biosciences | 50 | x |  |
| * CD15 BV510 only used in phagoBURST assay | | | | | | |

**Table S3. Phenotypic descriptions used to define B-cell subsets stained with EuroFlow PERISCOPE B-cell and plasma cell panel (BIGH) panel by manual analysis.** The removal of debris and doublets is not indicated in the analysis strategy below, but should be performed to ensure high quality data. This table was previously published as Supplemental Table in: Diks et al. Age and Primary Vaccination Background Influence the Plasma Cell Response to Pertussis Booster Vaccination. Vaccines, 2022.

| Stepwise approach (gating in 2D plots) | Phenotypic description |
| --- | --- |
| #1. Identification of total plasma cells | CD45+CD19dimCD38highCD21-CD24-  Light scatter properties are low/medium (between lymphocytes and monocytes). |
| #2. Definition of maturation stage | - Least mature plasma cells: CD20+CD138- - Intermediate mature plasma cells: CD20-CD138- - Most mature plasma cells: CD20-CD138+ |
| #3. Classification of plasma cells based on isotype | - IgM+, no expression of other isotype Igs - IgG1+, no expression of other isotype Igs - IgG2+, no expression of other isotype Igs - IgG3+, no expression of other isotype Igs - IgG4+, no expression of other isotype Igs - IgA1+, no expression of other isotype Igs - IgA2+, no expression of other isotype Igs - IgD+, no expression of other isotype Igs - IgH-, no Ig expression of any isotype Igs |
| #4. Classification of plasma cells based on CD62L expression | - CD62L- - CD62L+ |
| #5. Identification of total B cells | - CD45+CD19+CD20+ B cells show low light scatter characteristics (lymphocyte range) |
| #6. Identification of switched memory B-cell (MBC) subsets based on isotype. Switched MBCs express only one isotype | - IgG1+, no expression of other isotype Igs - IgG2+, no expression of other isotype Igs - IgG3+, no expression of other isotype Igs - IgG4+, no expression of other isotype Igs - IgA1+, no expression of other isotype Igs - IgA2+, no expression of other isotype Igs |
| #7. Subclassification based on maturation/functional CD markers | - CD20+CD21+   - Homogenous CD24 staining - CD20++CD21-/dim   - CD24+   - CD24- |
| #8. Subclassification based on CD62L/CD27 positivity | - CD27+CD62L+ - CD27+CD62L- - CD27-CD62L- - CD27-CD62L+ |
| #9. Identification of non-switched MBCs | CD27+IgM++IgD+  Of note, a minor subset of IgD+IgM- MBCs may be found as well. These can be classified separately. |
| #10. Subclassification based on maturation/functional CD markers | - CD20+CD21+   - Homogenous CD24 staining - CD20++CD21-/dim   - CD24+   - CD24-   No further subclassification in these populations. |
| #11. Classification of pre-germinal center (preGC) B cells | CD27-IgM+IgD+ |

| #12. Subclassification based on maturation/functional CD markers | - Immature preGC B cells: CD38+CD24+CD5+CD21-/+ - Naive CD5+ B cells: CD38-/dim CD24-/dimCD5+ - Naive CD5- B cells:CD38-/CD24-/dimCD5- |
| --- | --- |
| #13. Subclassification of naive B cells based on maturation/functional CD markers | - CD20+CD21+   - Homogenous CD24 staining - CD20++CD21-/dim   - CD24+   - CD24- |

**Table S4. Phenotypic descriptions used to define T-cell subsets stained with EuroFlow PERISCOPE CD4 T-cell (TCD4) panel by manual analysis.** The removal of debris and doublets is not indicated in the analysis strategy below, but should be performed to ensure high quality data. Official reference for the used panel and these phenotypic descriptions: Botafogo V, Pérez-Andres M, Jara-Acevedo M, et al. Age distribution of multiple functionally relevant subsets of CD4+ T cells in human blood using a standardized and validated 14-color EuroFlow immune monitoring tube. *Frontiers in Immunology.* 2020;11:166. And: Patent filed by Van Dongen et al. Means and Methods for Multiparameter Cytometry-Based Leukocyte Subsetting. P119646NL00 (2019). PCT/NL2020/050688, priority date 5 November 2019.

| Stepwise approach (gating in 2D plots) | Phenotypic description |
| --- | --- |
| #1 Identification of total CD4 T cells | CD3+CD4+CD45+  Light scatter properties are low (‘lymphocyte gate’). |
| #2 Identification of regulatory T cells (Tregs) within CD4 T cells | CD25+CD127dim |
| #3 Identification of follicular helper T cells (TFHs) within CD4 T cells | CD25-/dim CD185+ CCR10- |
| #4 Division of total CD4 T cells into T-helper (TH) subsets | - Divide based on chemokine receptor expression (CD183, CD194, CD196, and CCR10)   - Naive: CD27+CD45RA+CD62L+CD127+CD183-CD194-CD196-CCR10-   - TH1: CD183+ CD194-CD196-CCR10-   - TH2: CD183- CD194+CD196-CCR10-   - TH17: CD183- CD194+CD196+CCR10-   - TH1/17: CD183+CD194-CD196+CCR10-   - TH22: CD183- CD194+CD196+CCR10+   - CD183+CD194+CD196+CCR10+   - CD183+CD194+CD196+CCR10-   - CD183+CD194+CD196-CCR10+   - CD183+CD194+CD196-CCR10-   - CD183+CD194-CD196+CCR10+   - CD183+CD194-CD196-CCR10+   - CD183-CD194-CD196+CCR10-   - CD183-CD194+CD196-CCR10+   - Non-naive CD4+CD183-CD194-CD196-CCR10-CD27-/+CD45RA-CD62L-/+ |
| #5 Division of total Tregs in TH-like subsets | - Divide primarily based on chemokine receptor expression (CD183, CD194, CD196, and CCR10)   - Naive Treg: CD27+CD45RA+CD62L+ CD183-CD194-CD196-CCR10-   - TH1-like: CD183+CD194-CD196-CCR10-   - TH2-like: CD183-CD194+CD196-CCR10-   - TH17-like: CD183-CD194+CD196+CCR10-   - TH22-like: CD183-CD194+CD196+CCR10+   - CD183+CD194+CD196-CCR10+ Treg   - CD183+CD194+CD196-CCR10- Treg   - CD183+CD194+CD196+CCR10- Treg   - CD183+CD194+CD196+CCR10+ Treg   - CD183-CD194+CD196-CCR10+ Treg |
| #6 Division of total TFHs in Treg-/TH-like subsets | - Divide primarily based on chemokine receptor expression (CD183, CD194, CD196, and CCR10)   - CD185+CD27+CD45RA+CD62L+ T cells (CD183-CD194-CD196-CCR10-)   - Treg TFH: CD127+/dimCD183-/+CD194-/+CD196-/+CCR10-   - TH1-like: CD183+CD194-CD196-CCR10-   - TH2-like: CD183-CD194+CD196-CCR10-   - TH17-like: CD183-CD194+CD196+CCR10-   - TH1/17-like: CD183+CD194-CD196+CCR10-   - CD183+CD194+CD196-CCR10- TFH   - CD183+CD194+CD196+CCR10- TFH   - CD183-CD194-CD196+CCR10- TFH   - CD183+CD194+CD196-CCR10- TFH |
| #7 division of each subset into different maturation stage | - Divide based on CD27, CD45RA, and CD62L   - Central memory (CD27+CD45RA-CD62L+)   - Transitional memory (CD27+CD45RA-CD62L-)   - Effector memory (CD27-CD45RA-CD62L-/+)   - Terminal effector (CD27-CD45RA+CD62L-/+) |

**Table S5. Phenotypic descriptions used to define T-cell and NK-cell subsets stained with the EuroFlow PERISCOPE CD8 cytotoxic T-cell (CYTOX) panel by manual analysis.** The removal of debris and doublets is not indicated in the analysis strategy below but should be performed to ensure high quality data. Official reference for these phenotypic descriptions: Patent filed by Van Dongen et al. Means and Methods for Multiparameter Cytometry-Based Leukocyte Subsetting. P119646NL00 (2019). PCT/NL2020/050688, priority date 5 November 2019.

| Stepwise approach (gating in 2D plots) | Phenotypic description |
| --- | --- |
| #1 Identification of total T cells | CD3+CD45+  Light scatter properties are low (‘lymphocyte gate’). |
| #2 Identification of total TCRγδ+ T cells within total T cells | CD3+ TCRγδ+ |
| #3 Identification of total CD8+ and CD8- T cells within total T cells | CD8+ T cells: CD8+ TCRγδ-  CD8- T cells: CD8- TCRγδ- |
| #4 Identification of total NK cells | CD3- TCRγδ- CD45+  Light scatter properties are low (‘lymphocyte gate’).  CD56-/+  CD16-/+ (lower than neutrophils)  CD45RA-/+ (most NK cells are CD45RA positive)  CD62L-/+ |
| # 5 Identification of additional lymphocytes | Light scatter properties are low (‘lymphocyte gate’).  CD45+CD3-CD4- |
| #6 Identification of myeloid cells | Neutrophils: high SSC, CD16+CD45+  Eosinophils: high SSC, CD45+, Autofluorescence results in double positive population in CD57 vs cy Granzyme B plot  Monocytes: intermediate SSC, CD45+CD16+/- CD45RA-/+ and mostly CD62L+ |
| #7 Subsetting of TCRγδ+ T cells | Naive: CD27+CD28+CD45RA+CD62L+GranzB-CD57-  Central memory: CD27+CD28+CD45RA-CD62L+   - CD57-cyGranzB-/+ - CD57+CyGranzB+   Transitional memory: CD27+CD28-/+CD45RA-CD62L-/dim   - CD57-cyGranzB- - CD57-CyGranzB+ - CD57+CyGranzB+   Peripheral memory: CD27-CD28+CD45RA-CD62L-/+   - CD57-cyGranzB- - CD57-CyGranzB+ - CD57+CyGranzB+   Early effector: CD27+CD28-CD45RA+CD62L-/+   - CD57-CyGranzB+ - CD57+CyGranzB+   Terminal effector: CD27-CD28-CD45RA+CD62L-/+   - CD57-CyGranzB+ - CD57+CyGranzB+ |
| #8 Subsetting of CD8+ T cells | Naive: CD27+CD28+CD45RA+CD62L+ (NB: in some donors the naive population can be divided into CD62Lhigh and CD62Llow)  Central memory: CD27+CD28+CD45RA-CD62L+   - CD57-CyGranzB-/+ - CD57+ CyGranzB-/+   Transitional memory:CD27+CD28+CD45RA-CD62L-/dim   - CD57-cyGranzB- - CD57+CyGranzB- - CD57-CyGranzB+ - CD57+CyGranzB+   Peripheral memory: CD27-CD28-/+CD45RA-CD62L-/+   - CD57-cyGranzB- - CD57-CyGranzB+ - CD57+CyGranzB+   Early effector: CD27+CD28-CD45RA+CD62L-/+   - CD57-cyGranzB- - CD57-CyGranzB+ - CD57+CyGranzB+   Terminal effector: CD27-CD28-CD45RA+CD62L-/+   - CD57-cyGranzB- - CD57-CyGranzB+ - CD57+CyGranzB+ |
| #9 Subsetting of NK cells | CD56+ bright NK cells: CD56brightCD16lo/dim   - CD57-cyGranzB- - CD57-CyGranzB+   CD56+dim NK cells: CD56dimCD16+   - CD57-cyGranzB- - CD57-CyGranzB+ - CD57+CyGranzB+ |

**Table S6. Phenotypic descriptions used to define innate immune cell (sub)sets stained with the EuroFlow PERISCOPE DC-Monocyte panel by manual analysis.** The removal of debris and doublets is not indicated in the analysis strategy below, but should be performed to ensure high quality data. Official references for this phenotypic description: Van der Pan et al, Development of a standardized and validated flow cytometry approach for monitoring of innate myeloid immune cells in human blood, Frontiers in Immunology, 2022, 5141. And: Patent filed by Van Dongen et al. Means and Methods for Multiparameter Cytometry-Based Leukocyte Subsetting. P119646NL00 (2019). PCT/NL2020/050688, priority date 5 November 2019.

| Stepwise approach (gating in 2D plots) | Phenotypic description |
| --- | --- |
| #1 Identify eosinophils | SSC high, CD45+ neg. for all other markers in the panel |
| #2 Identify mature neutrophils | SSC high CD45+CD16+ |
| #3 Identify immature neutrophils | CD45+CD33+CD16-/+HLA DR-CD14-SLAN&FcER1-   - - CD62L-   - CD62L+ |
| #4 Identify monocytes | SSC intermediate  CD45+CD33+CD16-/+HLA DR+CD14-/+SLAN&FcER1-/+ |
| # 5 Divide the monocytes based on CD14/CD16 expression | ncMo: CD14-/dimCD16+CD62L- SLAN &FcER1-/+   - - SLAN+CD36+   - SLAN-CD36+   - SLAN-CD36-   - SLAN+CD36-   iMo:CD16+CD14+HLA DR+Slan&FcER1-CD300e&CD303+CD36+  cMo:CD16-CD14+CD62L-/+   - - CD62L+FcER1+   - CD62L-FcER1+   - CD62L+FcER1-   - CD62L-FcER1- |
| #6 Identify the CD1c+ myeloid DCs | SSC intermediate  CD45+CD33+CD141-/dimFcER1+HLA DR+CD16-CD14-/dim   - CD14dim - CD14- |
| #7 Identify the plasmacytoid DCs | SSC intermediate  CD45+CD303+CD14-HLA DR+CD16- |
| #8 Identify the CD141+ myeloid DCs | CD141+CD33+CD300e-CD303-CD14-HLA DR+CD16- |
| #9 Identify the Axl+ DCs within the plasmacytoid DCs | CD33dimCD141+CD36dim |
| #10 Identify the basophils | SSC intermediate  CD45dimCD33+CD303-CD300e-CD14-HLA DR-CD62L+ |
| # Identify ‘unspecified nucleated cells’ | Remaining CD45+ events that fit the singlet gate |

Table S8. Control and assay tubes measured in the phagocytosis experiment.

|  | **Wash** | **Stain** | **Stimulus** | **Inc. at 37°C** | **Lyse** |
| --- | --- | --- | --- | --- | --- |
| Control 1 | **+** | **-** | **-** | **-** | **+** |
| Control 2 | **+** | **+** | **-** | **-** | **+** |
| Control 3 | **+** | **+** | **-** | **+** | **+** |
| Control 4 (no cells, pHRodo™ Green *E. coli* bioparticles only) | **-** | **-** | **+** | **-** | **-** |
| Control 5 | **+** | **+** | **+** | **-** | **+** |
| Assay tube (n=2) | **+** | **+** | **+** | **+** | **+** |

Inc. = incubation of the sample, Lyse = lysis of erythrocytes

Table S9. Control and assay tubes measured when detection production of reactive oxygen species (ROS).

|  | **Wash** | **Stain** | **Stimulus** | **Inc. at 37°C** | **Lyse** |
| --- | --- | --- | --- | --- | --- |
| Control 1 | **+** | **-** | **-** | **-** | **+** |
| Control 2 | **+** | **+** | **-** | **+** | **+** |
| Control 3 (for FcR-dependent stim, n=2) | **+** | **+** | ***E. coli*** | **-** | **+** |
| Control 4 (for FcR-independent stim, n=2) | **+** | **+** | **PMA** | **-** | **+** |
| Assay tube (FcR-dependent, n=2) | **+** | **+** | ***E. coli*** | **+** | **+** |
| Assay tube (FcR-independent, n=2) | **+** | **+** | **PMA** | **+** | **+** |

Inc. = incubation of the sample, Lyse = lysis of erythrocytes, PMA = Phorbol 12-Myristate 13-acetate
